# Future redistribution of fishery resources suggests biological and economic trade-offs according to the severity of the emission scenario

**DOI:** 10.1371/journal.pone.0304718

**Published:** 2024-06-06

**Authors:** Irene D. Alabia, Jorge García Molinos, Takafumi Hirata, Daiju Narita, Toru Hirawake

**Affiliations:** 1 Arctic Research Center, Hokkaido University, Sapporo, Hokkaido, Japan; 2 Graduate School and College of Arts and Sciences, The University of Tokyo, Tokyo, Japan; 3 National Institute of Polar Research, The Graduate University for Advanced Studies, SOKENDAI, Tachikawa, Tokyo, Japan; Fisheries and Oceans Canada, CANADA

## Abstract

Climate change is anticipated to have long-term and pervasive effects on marine ecosystems, with cascading consequences to many ocean-reliant sectors. For the marine fisheries sector, these impacts can be further influenced by future socio-economic and political factors. This raises the need for robust projections to capture the range of potential biological and economic risks and opportunities posed by climate change to marine fisheries. Here, we project future changes in the abundance of eight commercially important fish and crab species in the eastern Bering Sea and Chukchi Sea under different CMIP6 Shared Socioeconomic Pathways (SSPs) leading to contrasting future (2021–2100) scenarios of warming, sea ice concentration, and net primary production. Our results revealed contrasting patterns of abundance and distribution changes across species, time periods and climate scenarios, highlighting potential winners and losers under future climate change. In particular, the least changes in future species abundance and distribution were observed under SSP126. However, under the extreme scenario (SSP585), projected Pacific cod and snow crab abundances increased and decreased, respectively, with concurrent zonal and meridional future shifts in their centers of gravity. Importantly, projected changes in species abundance suggest that fishing at the same distance from the current major port in the Bering Sea (i.e., Dutch Harbor) could yield declining catches for highly valuable fisheries (e.g., Pacific cod and snow crab) under SSP585. This is driven by strong decreases in future catches of highly valuable species despite minimal declines in maximum catch potential, which are dominated by less valuable taxa. Hence, our findings show that projected changes in abundance and shifting distributions could have important biological and economic impacts on the productivity of commercial and subsistence fisheries in the eastern Bering and Chukchi seas, with potential implications for the effective management of transboundary resources.

## Introduction

There is a growing recognition of the cumulative and synergistic impacts of climate change, fishing pressures, and poor or lacking management of marine fisheries resources, which are anticipated to be exacerbated in the future [1–3]. Marine wild-capture fisheries provide staple protein and nutrient sources for a large proportion of the world’s population and are among the significant drivers of economic development [4]. About 80 million metric tons of wild seafood are harvested from the ocean annually; a volume of protein comparable to global production of major land-based protein sources [5, 6]. In 2020, the global capture fisheries production had an approximate value of 141 billion USD and provided 390 million livelihoods depending, at least partially, on this fisheries sector [6]. Hence, climate-driven impacts to capture fisheries are expected to have cascading impacts on ocean-reliant sectors that support global and regional food security, human health, economic growth, and employment [7–9].

A significant portion of the Pacific Arctic region is comprised of the wide continental shelves of the eastern Bering Sea and Chukchi Sea (S1 Fig in S1 File). The former is one of the most productive ocean regions in the world, supporting a large biomass of economically-valuable fisheries resources [10]. Alaska fisheries harvests accounted for 60.26% of the total US commercial harvests and 32.09% of the US ex-vessel value in 2020 [11]. The groundfish fisheries in the Bering Sea-Aleutian Islands and Gulf of Alaska produce high levels of total catch (in metric ton, mt), ex-vessel revenues (US Dollar, USD), processed product revenues, exports, employment, and economic activity while ensuring the sustainability of fish stocks [10]. Ex-vessel prices correspond to the value received by fishers for their catches/harvests and are important for understanding fishing behavior dynamics [12]. Despite the effective management and extensive monitoring that are currently in place for these waters, climate change has resulted in ecological surprises such as the recent collapse of valuable fisheries resources (e.g. snow crab and Pacific cod) [13, 14], resulting in significant loss of livelihoods and revenue streams. In recent decades, the region has experienced pronounced climate-driven changes in thermal and sea ice conditions [15], primary productivity fluctuations [16, 17], and a redistribution of overall marine biodiversity [18, 19]. These oceanographic changes contributed to fluctuations in the abundance and distribution of commercially-important fisheries in these waters [20–23].

Given the large uncertainty about future climate outcomes and their impacts on marine fisheries [24], there is an urgent need for anticipating their potential bioeconomic consequences on marine resources using up-to-date climate scenarios to better inform proactive management strategies [25]. Traditionally, bioeconomic modeling in fisheries combines biological and economic parameters to identify appropriate levels of stock biomass and catch for resource management purposes [26]. In recent decades, the approach has been increasingly implemented to forecast global fishery prospects under different climate scenarios and fisheries management regimes [12, 27, 28]. These approaches are widely utilized to identify and map essential fish habitats [29, 30] and provide unbiased estimates of population trends that can be useful for stock assessment [31, 32]. Here, we adopt a bioeconomic model analysis to assess species-specific abundance changes of major fisheries resources in the Pacific Arctic and subsequent fluctuations in maximum catch, revenue, and abundance changes under future climate scenarios.

The main goal of this work is to examine changes in the projected abundance of a subset of commercially-important marine fish and invertebrate species in the eastern Bering Sea and Chukchi Sea using future climate and ocean productivity projections under four alternative Shared Socioeconomic Pathways (SSP) from the state-of-the-art climate models of the Coupled Model Intercomparison Project 6 (CMIP6) [33]. In doing so, we intend to capture taxon-specific responses under these divergent future socioeconomic and climate narratives and identify the potential ecological and economic implications of the changes in distributions and abundances of eight commercially-important species, which together accounted for over 65% of the total landings in the Bering Sea-Aleutian Islands between 2000 and 2019 [10, 34].

## Materials and methods

### Physical and biogeochemical data

The present-day climate data at 0.25° x 0.25° spatial resolution and bathymetry for the eastern Bering and Chukchi seas were downloaded from online data repositories for the years 2000 to 2019. Daily data of the Advanced Very High-Resolution Radiometer-Optimally Interpolated (AVHRR-OI) sea surface temperature (SST,°C) and sea ice concentration (SIC, %) were downloaded from the National Centers for Environmental Observations (NCEI) of the National Oceanic and Atmospheric Administration (NOAA; https://www.ncei.noaa.gov/data/sea-surface-temperature-optimum-interpolation/v2.1/access/avhrr/; date accessed: 06 July 2021). This period exhibited record-breaking changes in temperature and sea ice in the Arctic and surrounding marine areas [19, 35]. From the daily data, we computed the average winter SST (WSST) and sea ice concentration (WSIC). In this work, winter was defined as January to April and summer as June to August. Seasonal sea bottom temperatures for summer (SSBT) and winter (WSBT) from the Global Ocean Ensemble Physics Reanalysis were downloaded via the Copernicus Marine Environment Monitoring Service (CMEMS; https://marine.copernicus.eu/, date accessed: 24 November 2022). Summer net primary production (SNPP) was computed from monthly model outputs of the Global Ocean Biogeochemistry hindcast, downloaded from CMEMS (https://data.marine.copernicus.eu; date accessed: 22 November 2022). We used the ETOPO1 data from one arc-minute global relief model of the Earth’s surface integrating land topography and ocean bathymetry [36], downloaded from the NOAA NCEI online repository (https://www.ngdc.noaa.gov/mgg/global/; date accessed: 08 July 2021). These environmental variables were selected as model covariates based on their importance on species distributions and abundance in the study area [19, 20].

The future climate (WSIC, WSST, WSBT, and SSBT) and primary production (SNPP) layers were calculated from the corresponding monthly averages of four down-scaled CMIP6 Global Climate Models (GCMs; CESM2-WACCM, CNRM-ESM2-1, IPSL-CM6ALR, and MPI-ESM1.2HR). These GCMs were used for this study as they were shown to have good agreement with the Arctic sea ice extent observations [37]. First, the monthly-resolved historical (1850–2014) and projected (2015–2100) climate data were downloaded from the Earth System Grid Federation (ESGF) CMIP6 online repository (https://aims2.llnl.gov/search; date accessed: 15 September 2022). These included four Shared Socio-economic Pathways (SSP126, SSP245, SSP370, and SSP585) from the new climate scenario framework established by the climate change research community to promote the integrated analysis of future climate change impacts, vulnerabilities, adaptation, and mitigation [38]. These pathways are based on the narratives describing the alternative socio-economic developments and specific radiative forcing levels, analogous to the representative concentration pathways (RCPs), at the end of the 21^st^ century: (i) sustainable development at 2.6 W m^-2^, SSP126; (ii) middle of the road at 4.5 W m^-2^, SSP245; (iii) regional rivalry at 7.0 W m^-2^, SSP370; and (iv) fossil-fueled development at 8.5 W m^-2^, SSP585 [39]. Finally, we implemented the delta method for spatial data down-scaling of future climate layers by computing the pixel-wise difference between the historical present (2000–2014) and future (2021–2100) coarsely-resolved (1° x 1°) values of averaged climatic and productivity variables across the four GCMs for each SSP. Each difference raster was then resampled to 0.25° x 0.25° resolution and added to present-day (2000–2019) satellite and biogeochemical model hindcast and reanalysis data of similar spatial resolution [40].

### Species abundance data

Abundance data (expressed as catch per unit effort, CPUE, in kg/km^2^) of eight commercially-important species in the eastern Bering Sea were downloaded from the US NOAA Fisheries online repository (https://www.fisheries.noaa.gov/foss/; date accessed: 24 November 2022). Summer (June-August) bottom trawl surveys conducted over the southeastern Bering Sea were available between 2000 and 2019. However, bottom trawl surveys in the northeastern Bering Sea were only conducted in 2010, 2017 and 2019. Available species abundance data in the US Chukchi Sea area from the 2012 Arctic ecosystem integrated survey (August-September) were also compiled for the target taxa (Arctic Eis Program) [41]. This subset of marine species represents some of the major fisheries in terms of landings and economic values for Alaska (https://www.fisheries.noaa.gov/foss) during the last two decades (2000–2019; S1 Table in S1 File).

### Species abundance model construction

Machine learning algorithms often outperform conventional regression models in predicting species distribution and abundance, as the former are able to better capture complexity and are more flexible relative to the latter [42, 43]. Here, we used annually compiled species-specific abundance, climate layers (WSIC, WSST, WSBT, and SSBT), SNPP, and bathymetry to develop two machine-learning (random forest, RF; and boosted regression trees, BRT) models to predict the present and projected future patterns of species abundance in response to ongoing and anticipated climate and productivity changes. These machine learning algorithms both handle continuous data as model response variables. In particular, the random forest (RF) model is an “ensemble learning” approach that combines large aggregations of decision trees to reduce the variance relative to a single decision tree [44, 45]. The boosted regression trees (BRT) model is likewise based on an ensemble method that takes advantage of strengths of regression trees and boosting algorithms. While the RF model relates a response to predictors by iterative binary splits, the BRT model implements an adaptive approach that combines a large number of simple models to generate improved predictive performance [46, 47].

In implementing machine learning algorithms, balancing the model complexity and parsimony is critical to achieving the best model predictions while avoiding overfitting [48]. This is done by tuning the models’ hyperparameters (i.e., parameters with fixed values that must be defined before model training), with unknown optimal values which are often specific to the modeling problem and dataset [49, 50]. Hence, we tuned the hyperparameters of species-specific RF models (i.e., number of features to split at each node, minimum node size, and number of trees) and BRT models (i.e., learning rate, interaction depth, and number of trees) to optimize model complexity and performance in predicting abundance. The tuning process resulted in a total of 2700 initial runs for each species and obtained optimal hyperparameters based on the minimum Root-Mean-Squared Error (RMSE) in the predicted species abundance (S2, S3 Tables in S1 File). Using the optimal hyperparameter values, the final species-specific RF and BRT models were then trained and evaluated using 80% and 20% of the species observations, respectively. Species-specific RF models were tuned and built using the ‘ranger’ package version 0.14.1 [51] while BRT models were implemented using the ‘gbm’ package version 2.1.8.1 [52] in the R software version 4.2.2 [53]. The final models were then used to generate present predictions and future projections of species abundances using averaged climatic and productivity layers for present (2000–2019) and future periods (i.e., 2021–2040, 2041–2060, 2061–2080, and 2081–2100) under four alternative shared socio-economic pathways (i.e., SSP126, SSP245, SSP370, and SSP585). The ensemble model predictions/projections of species abundance were subsequently computed as the weighted average of RF and BRT, with the former models given higher weights based on lower RMSEs in predicted abundance for all species relative to the latter (S2, S3 Tables in S1 File). Given that the study area is conservatively managed (see next section), the potential impact of fishing on carrying capacity is potentially kept to a minimum. Hence, we assumed that predicted present-day (2000–2019) species abundances are at their carrying capacity, and that changes in projected abundances (2021–2100) reflect changes in fish biomass in response to combined fluctuations in future climate and primary productivity. However, it is important to note that other factors unaccounted in this analysis can influence species biomass and carrying capacity of an ecosystem. For instance, limiting factors such as biotic interactions (e.g., competition and predation pressure) and diseases are known to impact species biomass and carrying capacity [54].

### Biological and economic implications

#### Biological variables

The eastern Bering Sea (EBS) is managed under a precautionary approach and covers the Bering Sea-Aleutian Island (BSAI) management area within the US Exclusive Economic Zone [55]. This limits the optimum yield of the BSAI groundfish complex to 85% of a historical estimate of maximum sustainable yield (MSY), or between 1.4 and 2.0 million metric ton [55]. The target species in the groundfish complex is comprised of 19 taxon groups [55] and seven of the major species were included in this analysis. In contrast, BSAI King and Tanner (*Chionoecetes*) crabs are managed using a five-tier system that accommodates different levels of uncertainty of information [56]. For the individual crab fishery, the optimum yield ranges from 0 to less than the overfishing level (OFL) catch. For crab stocks, OFL refers to the annualized MSY, derived through the yearly assessment process [56]. While we recognize the importance of these management approaches in supporting the sustainable resource harvesting in the BSAI, our present exercises relied on constant species-specific biological parameters (e.g., population growth rate, fishing mortality rate, and scaling parameter of the yield curve; S4 Table in S1 File) from long-term averages of historical reference based on the RAM Legacy Stock Assessment Database version 2.95 [57] used by and extracted from previous studies [12, 58]. This database contained 504 stock assessments, which provided time series of harvest and reference values for the subset of species in the BSAI used for this study. Species-specific reference values were also standardized to a common baseline year (2012) to align the different time frames of the available data. The common baseline year is within the present-day (2000–2019) time period used for this work, thus, provide reasonable estimates of biological reference for each species in the study area. Our analyses further assumed predicted and projected species abundances based on survey data provide reasonable indices of fishable biomass [59]. These assumptions were necessary given that our species abundance models did not incorporate population age or size structure, or account for sampling survey selectivity, and such precludes the estimation of meaningful future biological parameters.

To explore biological and economic implications of climate-driven species abundance changes across time periods and contrasting socioeconomic pathways, we first calculated the maximum sustainable yield (MSY) using model outputs of species-specific abundance within the US exclusive economic zone (EEZ) of the EBS. This parameter pertains to the highest theoretical yield at equilibrium that can be continuously taken from a fish stock under the prevailing environmental conditions while allowing the population to sustain itself [5]. Here, we applied the historical biological reference values of species-specific population growth rate and scaling parameter (S4 Table in S1 File) to estimate the MSY of each species stock in the Bering Sea at each 20-year time-period using the following equation [58]:

$$MSY=\frac{gB}{{\left(\emptyset +1\right)}^{1/\emptyset }}$$ (1)

where *g* is the estimate of the species-specific population growth rate derived as *MSY*/*B*_*MSY*_ based on their reference baseline status (year 2012) [58], *B* is the species abundance predicted/projected from ensemble models, ∅ is the scaling parameter of the Pella-Tomlinson yield curve, which sets the ratio of the $\frac{B_{MSY}}{K}$ equal to 0.40 (∅ = 0.188) [60] and 0.35 (∅ = −0.093) [56] for groundfish and snow crab in the BSAI, respectively.

We then computed the maximum catch potential (MCP), which is also considered a proxy for the maximum sustainable yield (MSY) [61, 62] for each species stock in the EBS and Chukchi Sea given the following equation:

$$MCP=B*U_{MSY}$$ (2)

where *B* is the model-derived species biomass and *U*_*MSY*_ is the reference exploitation rate at maximum sustainable yield extracted from Gaines et al. [58]. Here, *U*_*MSY*_ represented the standardized species-specific exploitation rate at MSY (*U*_*MSY*_) expressed as the ratio of annual catch and biomass (*C*/*B*) for each of the BSAI species stock.

We assumed the maximum catch for each species is within a healthy level of exploitation (*U*_*MSY*_). For the present-day period (2000–2019) and future periods (2021–2100), MCP for each species was computed using the predicted and projected abundance (*B*) and reference *U*_*MSY*_ extracted from Gaines et al. [58]. Reference *U*_*MSY*_ for each species stock was calculated from annual exploitation rates at MSY in the BSAI, standardized to a common baseline year (2012) and assumed to remain constant in the future. As data timeframes for each species varied between 36- and 61-year periods (1949–2013), all stocks ending before 2012 were extended to this period using the same data from the most recently available year for standardization, centering the 2012 parameter value around the mean with a unit of standard deviation [12].

#### Economic variables

To obtain a simple assessment of the economic impacts of potential abundance changes, our subsequent analyses assumed constant prices and costs over time, as changes in their values are inherently difficult to forecast [58]. However, we recognized that these parameters could vary in the future due to other factors unaccounted for such as the potential changes in subsidies to fishing sectors and global fuel prices that can increase fishing costs [63]. Similarly, future prices are subject to change with potential increases in the global supply through aquaculture [64] and demand for seafood with concomitant changes in welfare and income [65].

For this exercise, we computed the revenue (*R*) and profit (*π*) for each species and for all of species combined. Firstly, we computed species-specific revenue, which refers generally to the income generated from harvesting fish and was obtained using the equation:

R=p*MCP (3)

where *p* is the ex-vessel price for each fishery in year 2012 expressed in constant US Dollar/metric ton (USD/mt) extracted from Gaines et al. [58] and were based on FAO export data and published estimates of average ex-vessel values [12]. Global economic parameters used in this study were earlier compared with prices obtained from fishery management agencies in different countries for each species showing general reasonable approximations of true ex-vessel prices [12]. *MCP* is the maximum catch potential of each taxon. Accordingly, we computed for the maximum revenue potential (*MRP*) which is defined as the total potential income from the catches of all species stocks (*n* = 8), derived using the following equation from Lam et al. [62]:

$$MRP=\sum_{i=1}^{n}\left(p_{i}∙{MCP}_{i}\right)$$ (4)

where *p*_*i*_ is the ex-vessel price and *MCP*_*i*_ is the maximum catch potential of species *i* and *n* is the total number of species.

Prior to calculating the profit (*π*), we first derived the cost parameter per unit of fishing mortality (*c*) using the following equation [12, 58]:

$$c=\frac{p\bar{fb}MSY}{{\left(g\bar{f}\right)}^{\beta }}$$ (5)

where *p* is the ex-vessel price weighted by the *MSY* $\bar{f}$ is (*F*/*F*_*MSY*_) when the fishery is at bioeconomic equlibrium, $\bar{b}$ is *B*/*B*_*MSY*_, *g* is the population growth parameter, and *β* is the non-linear fishing cost constant set at 1.3. *β* is a parameter that regulates the non-linearity of cost, where *β* >1 suggests that the cost per unit effort increases with each added unit [12, 58]. Species-specific historical reference values of $\bar{f},\bar{b}$, and *g* (S4 Table in S1 File) were similarly extracted from Gaines et al. [58]. We subsequently estimated the profit at MSY (*π*_*MSY*_) for individual species using the following equation:

$$\pi_{MSY}=R-cg^{\beta }$$ (6)

where species-specific parameters for *R*, *c*, *g*, and *β* were defined from the previous equations.

Consequently, the maximum profit potential (MPP) for all harvested species at a given time period and shared socioeconomic pathways was computed as the total profit from all eight species (*n*) following the equation:

$$MPP=\sum_{i=1}^{n}\left({\pi_{MSY}}_{i}\right)$$ (7)

where ${\pi_{MSY}}_{i}$ is the profit obtained from the harvest of species *i* and *n* is the total number of species.

To better account for the inherent uncertainty of our economic estimates, we conducted sensitivity analysis to examine how the choice of prices and costs influence our main results under three alternative scenarios [58]: (i) increasing costs, where prices remain constant while costs increase by 20% at a constant rate; (ii) increasing prices, where costs remain constant while prices increase by 20% at a constant rate; (iii) increasing costs and prices increase by 20% at a constant rate. These scenarios assumed costs and/or prices varying at a constant rate over time reaching a 20% increase at the end of the projection period (i.e., 2100) [58]. Overall, results of the sensitivity analyses showed similar trends in economic variables, although their magnitude varied slightly compared to the original constant prices and costs scenario (S5 Table and S1 Fig in S1 File). These results suggest that our findings are robust to the different assumptions on prices and costs over time.

#### Potential biological and economic impacts

Using the derived bioeconomic variables at each time period and SSPs, we examined bioeconomic implications of future species abundance fluctuations throughout the study area and computed the future percent changes in total MCP, MRP, and MPP relative to the present. We also quantified changes in the MCP and MRP at each time period and SSPs by averaging values at 100-km buffer area away from the major US fishing (i.e., Dutch Harbor in the south) and Arctic ports (Port of Nome in the north; S2 Fig in S1 File). For this analysis, we only focused on the total MCPs within the US territorial waters of the Eastern Bering and Chukchi seas.

We also explored the bioeconomic changes between the present-day fishing grounds within the US and Russian waters of the Eastern Bering Sea to examine the potential impact of future shifts of marine resources to fishing fleet dynamics. BSAI bioeconomic parameters for each species were used at both fishing grounds as these parameters were currently unavailable in Russian EEZ. Here, present-day fishing grounds were identified as pixels of high aggregated fishing effort over the last nine years (≥ 1000 fishing hrs) using annually aggregated fishing effort (in total fishing hours) available from 2012 to 2020 from the global fishing watch online repository (https://globalfishingwatch.org/data-download/datasets/public-fishing-effort; date accessed: 02 February 2023).

## Results

### Spatial distributions of physical and biogeochemical variables

**Fig 1:**
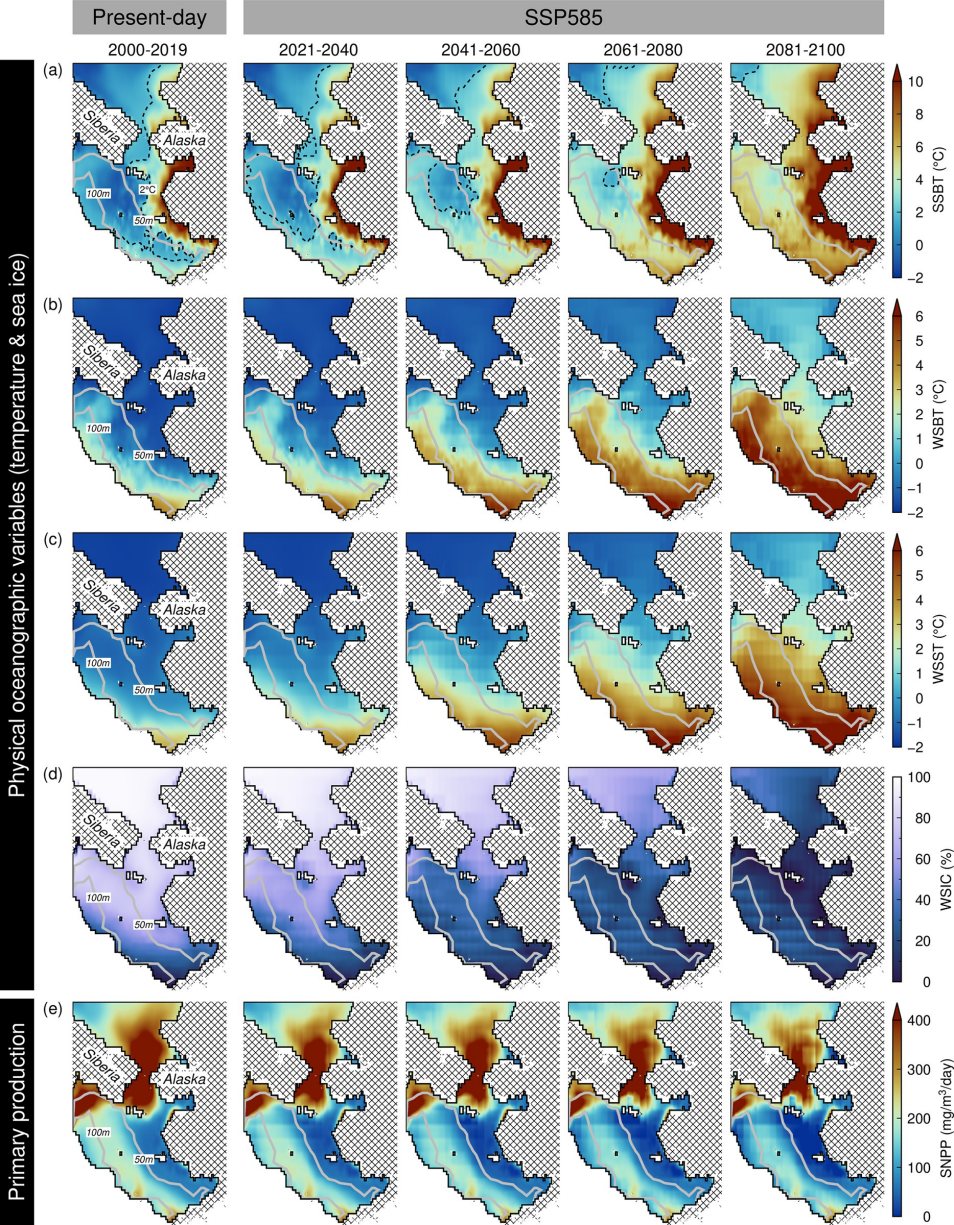
Spatial distributions of (a) summer sea bottom temperature (SSBT), (b) winter sea bottom temperature (WSBT), (c) winter sea surface temperature (WSST), (d) winter sea ice concentration (WSIC), and (e) summer net primary production (SNPP) between present (2000–2019) and future periods (2021–2100) under the SSP585 scenario (see S3-S5 Figs in S1 File for the other scenarios). Overlain on (a) is the cold pool feature (SSBT < 2°C; dashed lines) and bathymetric contours (solid gray lines).

Physical variables and primary production showed remarkable changes between the present and future periods under the different scenarios, with a particularly prominent contrast under the highest warming scenario (SSP585; Fig 1 and S3-S5 Figs in S1 File). In particular, the region covered by the cold pool feature (SSBT ≤ 2°C) in the EBS declined and disappeared during 2021–2040 and 2081–2100, respectively (Fig 1A). These patterns are also accompanied by the warming of the winter sea bottom and surface temperatures (Fig 1B and 1C), and the extensive loss of the winter sea ice (Fig 1D). The future summer integrated primary production also declined relative to the present (Fig 1E), with peaks situated around the Bering Strait. The enhanced primary production in these waters, however, is also projected to decrease progressively over time and with increasing severity of the SSPs (Fig 1E and S3-S5 Figs in S1 File). However, the waters in the southeastern Bering Sea (≤ 60°N; SEBS) showed future primary production at levels comparable to the present. For majority of the taxa, environmental variables including depth, bottom temperatures (SSBT and WSBT), and summer net primary production (SNPP), were the most important environmental factors influencing species distribution and abundance (S6, S7 Tables in S1 File). Overall, species-specific models were able to account between 55% and 87% of the variance in species abundance from 2000–2019 (S2, S3 Tables in S1 File).

### Spatial patterns of species-specific abundance

**Fig 2:**
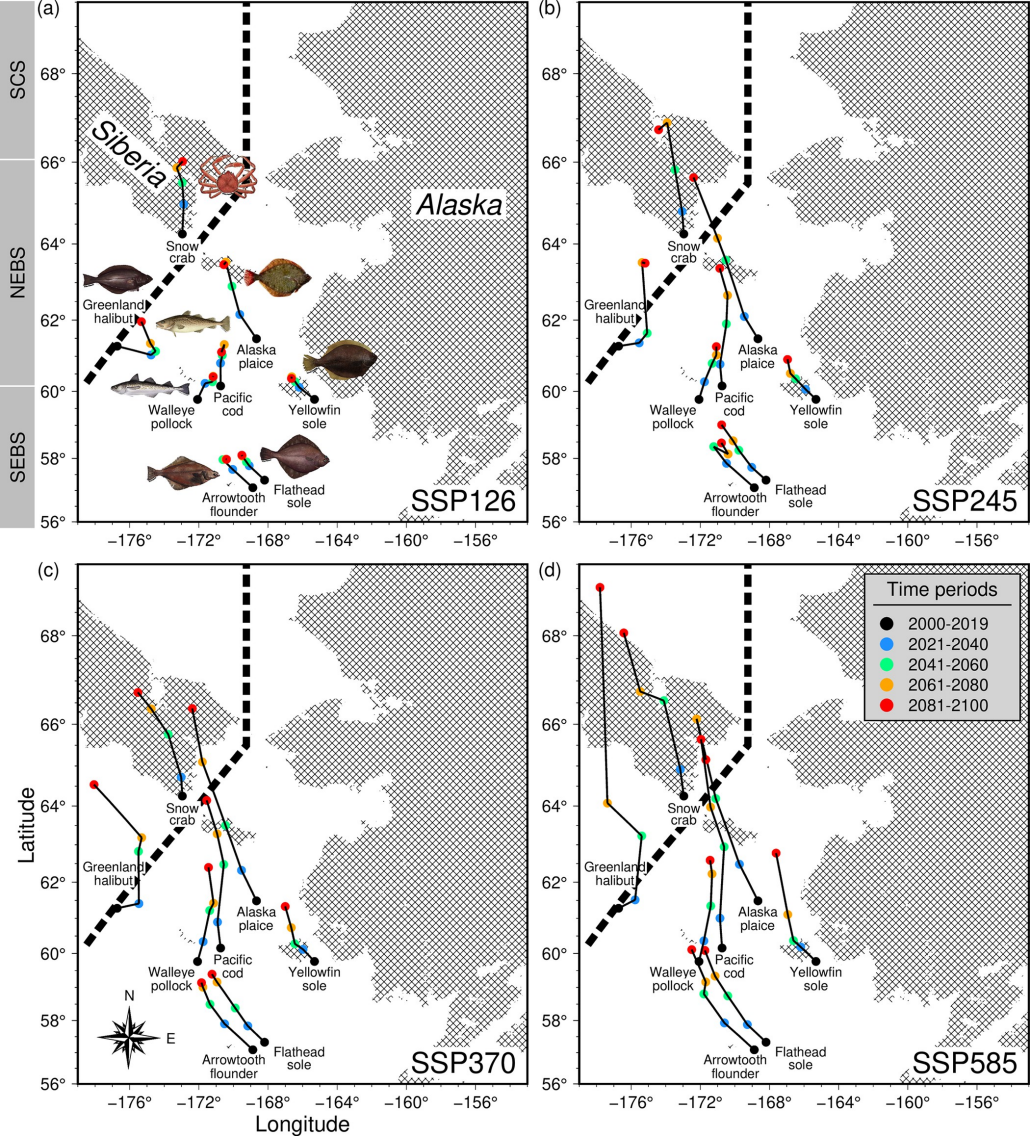
Trajectories of abundance-weighted center of gravity for eight (COGs) commercial species from modeled abundance from the present (2000–2019) to future periods (2021–2100) under the (a) SSP126, (b) SSP245, (c) SSP370, and (d) SSP585 scenarios. Overlain on the maps are the land masses (hachure lines) for reference and dashed lines correspond to the US-Russia exclusive economic zone (EEZ) boundary. As the taxon-specific COGs were computed throughout the basin separated by land masses, some trajectories were mapped on land for species with abundances close to the coasts (e.g., snow crab, yellowfin sole, Greenland halibut, and Alaska plaice). Species photo credits (https://www.fisheries.noaa.gov/species/).

At present, half of the species pool has their center of gravity (COG) of abundance situated in the SEBS (≤ 60°N; Fig 2A–2D). However, under the future conditions and at varying magnitudes of climate change, the COGs of abundance for all species are projected to shift northwards. This is accompanied by zonal displacements towards shallower waters of the middle domain of the northeastern Bering Sea (NEBS), particularly for the walleye pollock and Pacific cod, with large signals observed from the middle to late century periods under intermediate to high shared socioeconomic pathways (Fig 2B–2D). Overall, the highest rates of shift in the COG of abundance relative to the present (2000–2019) are projected for the late-century (2076–2100) period under the SSP585 scenario for the Greenland halibut (> 80 km/decade), and the lowest for the walleye pollock (31.64 km/decade; Table 1). Large shifts in the COG of abundance for majority of the species resulted from an extensive loss of optimal habitat due to significant sea ice loss and warming in the EBS under the extreme shared socio-economic pathway (SSP585; Fig 1 and S8 Table in S1 File). With respect to the present-day fishing grounds within the US and Russian waters, the COG of abundance for most species were located within the SEBS (S6 Fig in S1 File) and waters close to the US-Russian EEZ border (S7 Fig in S1 File), respectively. Under different climate scenarios, the COG of abundance also showed substantial spatial shifts across taxa resulting in an increasing overlap in areas of peak abundance within the present-day fishing grounds in both countries (S6, S7 Figs in S1 File).

**Table 1:** Species-specific rates of the shift in the abundance-weighted center of gravity (COG; km/decade) across future time periods (2021–2100) relative to the present (2000–2019) under the different shared socioeconomic pathways (SSPs).

Scenario	Species	Time periods			
		2021–2040	2041–2060	2061–2080	2081–2100
SSP126	Walleye pollock	14.32	12.40	11.25	8.83
	Pacific cod	18.14	16.18	16.39	10.71
	Yellowfin sole	15.80	14.13	12.87	10.00
	Arrowtooth flounder	23.66	23.64	16.97	13.38
	Snow crab	20.47	23.37	22.58	19.65
	Flathead sole	18.17	14.75	14.12	11.60
	Alaska plaice	22.40	28.98	30.69	24.12
	Greenland halibut	27.18	20.05	12.89	10.55
SSP245	Walleye pollock	14.90	20.46	18.94	17.64
	Pacific cod	17.36	32.38	35.01	35.85
	Yellowfin sole	11.69	15.52	14.61	15.63
	Arrowtooth flounder	31.99	33.09	18.39	19.07
	Snow crab	15.71	29.37	37.44	28.63
	Flathead sole	16.62	23.12	21.94	24.07
	Alaska plaice	19.86	42.07	39.84	49.69
	Greenland halibut	16.12	16.10	32.41	26.02
SSP370	Walleye pollock	16.63	27.75	23.93	29.51
	Pacific cod	20.77	43.13	43.59	44.60
	Yellowfin sole	13.47	14.20	16.38	19.75
	Arrowtooth flounder	33.20	35.74	34.15	28.74
	Snow crab	13.12	28.74	31.12	30.10
	Flathead sole	20.16	25.73	32.51	29.04
	Alaska plaice	25.82	40.40	53.87	57.25
	Greenland halibut	17.14	30.60	28.04	36.88
SSP585	Walleye pollock	17.10	29.99	34.59	31.64
	Pacific cod	23.72	51.81	53.31	61.38
	Yellowfin sole	16.95	16.41	21.75	35.72
	Arrowtooth flounder	34.73	42.89	35.47	39.63
	Snow crab	18.61	43.56	37.80	45.18
	Flathead sole	22.16	34.16	35.29	36.88
	Alaska plaice	31.05	54.22	68.08	43.47
	Greenland halibut	14.27	37.97	39.07	86.34

**Fig 3:**
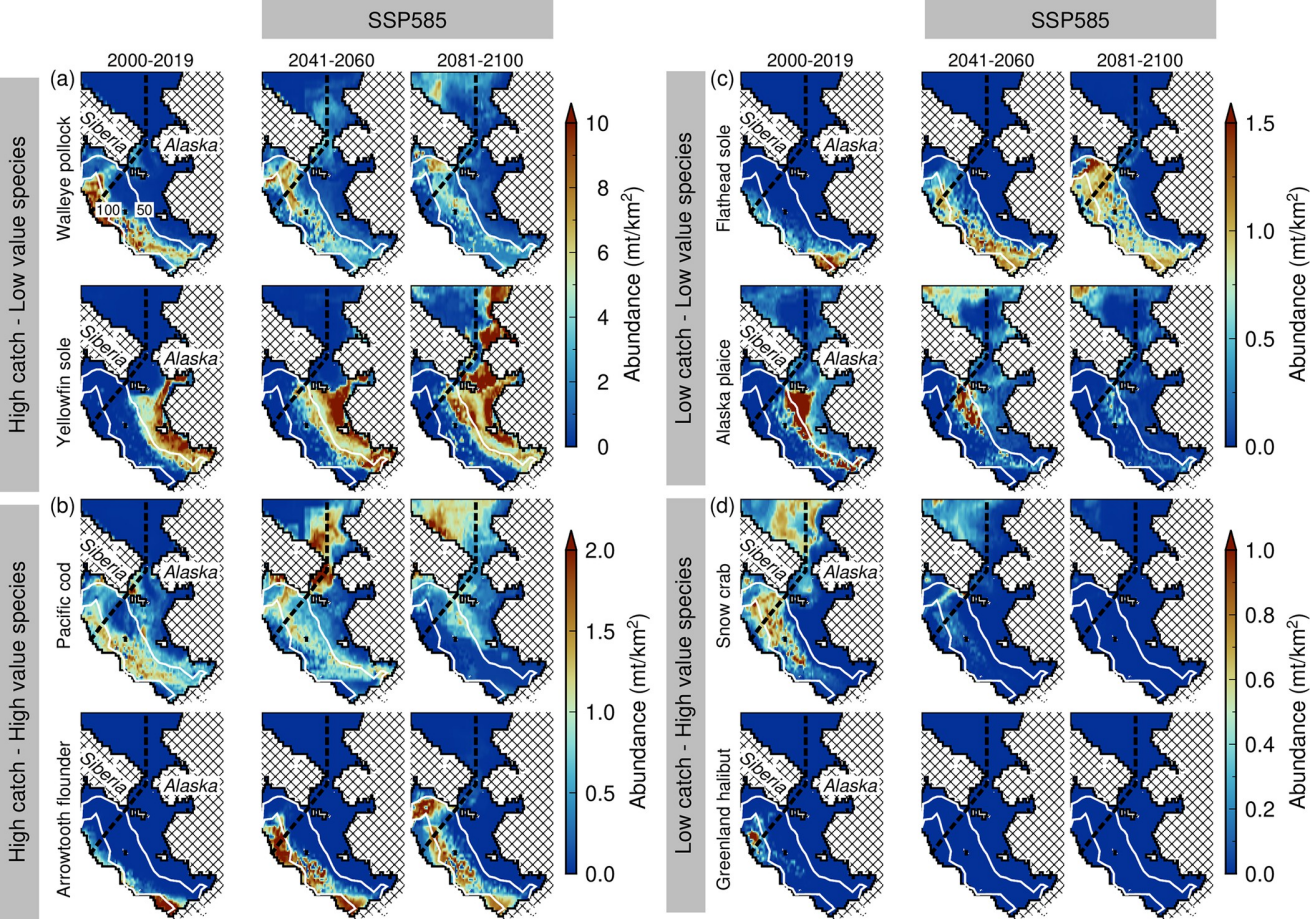
Spatial abundance distributions of (a) high catch–low value, (b) high catch–high value, (c) low catch–low value, and (d) low catch–low value species for present (2000–2019) and future (2041–2060; 2081–2100) under the SSP585 scenario. Overlain are the US-Russia EEZ boundary (dashed lines) and bathymetric contours (solid white lines).

The spatial distributions of species-specific abundance similarly reveal spatial changes proportional to time periods and SSPs (Fig 3 and S8 Fig in S1 File). In particular, the highest contrast in the present and future abundances for representative taxa, categorized based on their reported catch and economic values was observed under the SSP585 scenario (Fig 3A–3D). For abundant cod (walleye pollock and Pacific cod; Fig 3A) and flatfish taxa (yellowfin sole; Fig 3B, top panel), apparent future abundance declines were observed in the EBS and increases in the Chukchi Sea. In contrast, arrowtooth flounder (Fig 3B, bottom panel) showed future abundance increase limited only to the EBS. For low catch taxa (Fig 3C and 3D), abundance changes revealed opposite patterns between low (flathead sole and Alaska plaice; Fig 3C) and high (snow crab and Greenland halibut; Fig 3D) economic value species. The latter is characterized by the potential abundance declines and subsequent collapse of these high-value species in the EBS, while the low-value species exhibited substantial increases in the middle domain of the EBS (flathead sole) and Chukchi Sea (Alaska plaice) between 2041 and 2100.

### Changes in maximum catch, revenue, and profit potential

**Fig 4:**
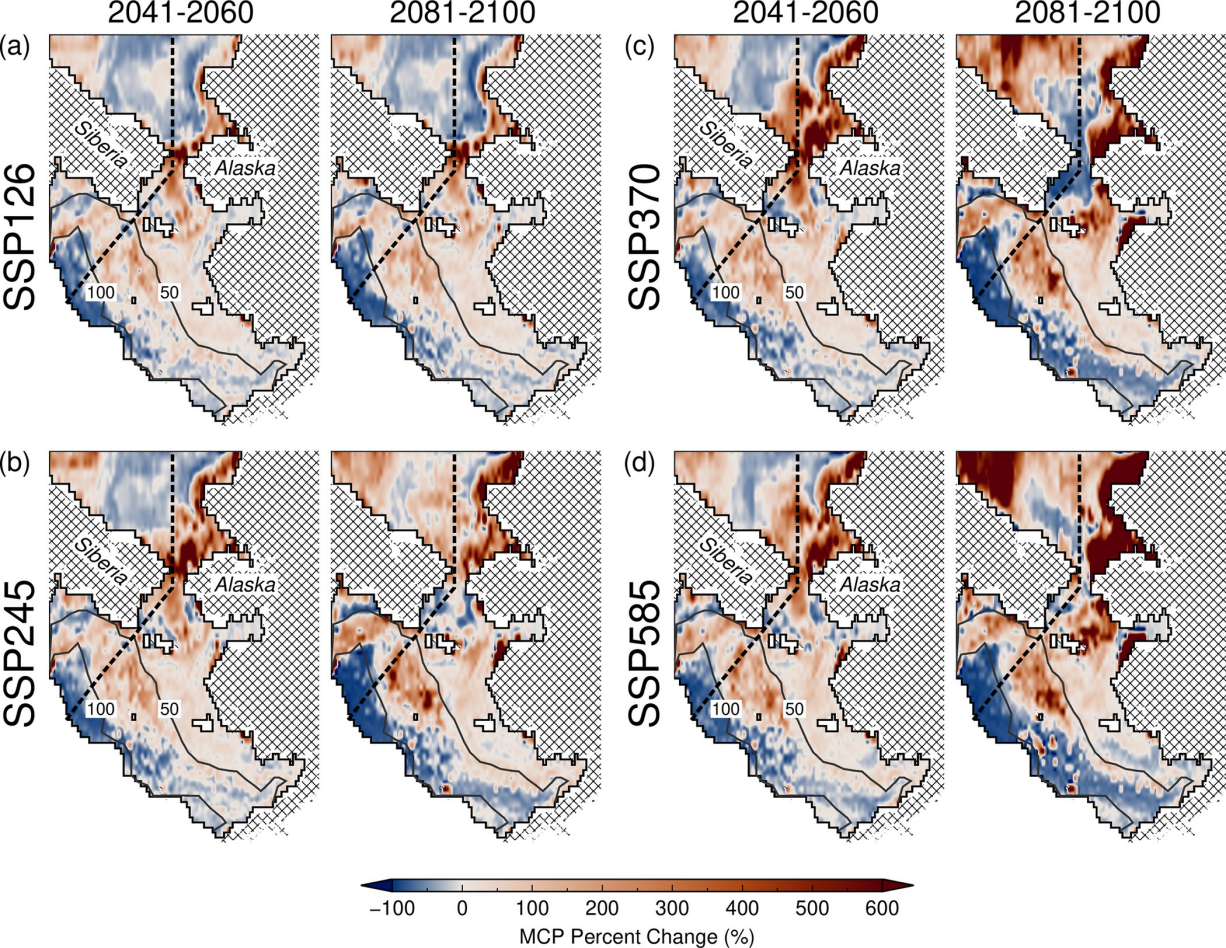
Predicted future (2041–2060; 2081–2100) percent changes in the cumulative maximum catch potential (MCP) under (a) SSP126, (b) SSP245, and (c) SSP370, and (d) SSP585 for eight major fisheries relative to the present (2000–2019). Overlain are the US-Russia EEZ boundary (dashed line) and bathymetric contours (solid gray lines).

Spatial distribution of differences in cumulative maximum catch potential (MCP) for the eight fisheries between the present and future periods also revealed large changes across specific time periods and climate scenarios, showing a tendency to increase with scenario severity and longer projected time horizons (Fig 4 and S9 Fig in S1 File). In particular, projected changes in cumulative maximum catch potential were lowest under SSP126 (Fig 4A), moderate under SSP245 (Fig 4B) and SSP370 (Fig 4C), and highest under SSP585 (Fig 4D). Significant MCP increases in the Chukchi Sea were accounted for by increases in the abundance of high catch and low economic value taxa (i.e., yellowfin sole and walleye pollock, S8A Fig in S1 File). The basin-wide percent differences in MCP, MRP, and maximum profit potential (MPP) relative to the present-day period (2000–2019) were also highest during the late-century (2081–2100) under SSP585 (Table 2). During this period and SSP, MCP decreased minimally by 4.22%, as major abundance declines are compensated by the abundance increases of other flatfish species (e.g., yellowfin sole, arrowtooth flounder, and flathead sole; S8 Table in S1 File). Nonetheless, this small abundance decline translated to significant drops in MRP and MPP by about 37% and 47%, respectively. This was due to reduction and shift in abundance of low-catch and high-economic value species (e.g., snow crab and Greenland halibut; S8D Fig and S8 Table in S1 File).

**Table 2:** Percentage of change in the maximum catch potential (MCP), maximum revenue potential (MRP), and maximum profit potential (MPP) relative to present (2000–2019) across different time periods (2021–2100) and contrasting shared socioeconomic pathways (SSPs) throughout the study area.

Scenario	Variable	Time periods			
		2021–2040	2041–2060	2061–2080	2081–2100
SSP126	MCP	1.92	-4.55	-5.27	-7.10
	MRP	-12.02	-18.28	-20.92	-23.12
	MPP	-15.72	-22.74	-26.21	-28.25
SSP245	MCP	4.68	-0.86	-3.65	-7.09
	MRP	-6.7	-17.42	-23.88	-32.55
	MPP	-9.95	-23.3	-30.77	-40.55
SSP370	MCP	5.58	2.8	-3.22	-5.65
	MRP	-6.72	-16.81	-29.07	-35.5
	MPP	-10.38	-23.46	-37.66	-44.28
SSP585	MCP	3.98	-2.04	-3.89	-4.22
	MRP	-9.27	-23.96	-33.79	-37.37
	MPP	-13.22	-31.66	-42.68	-46.83

**Fig 5:**
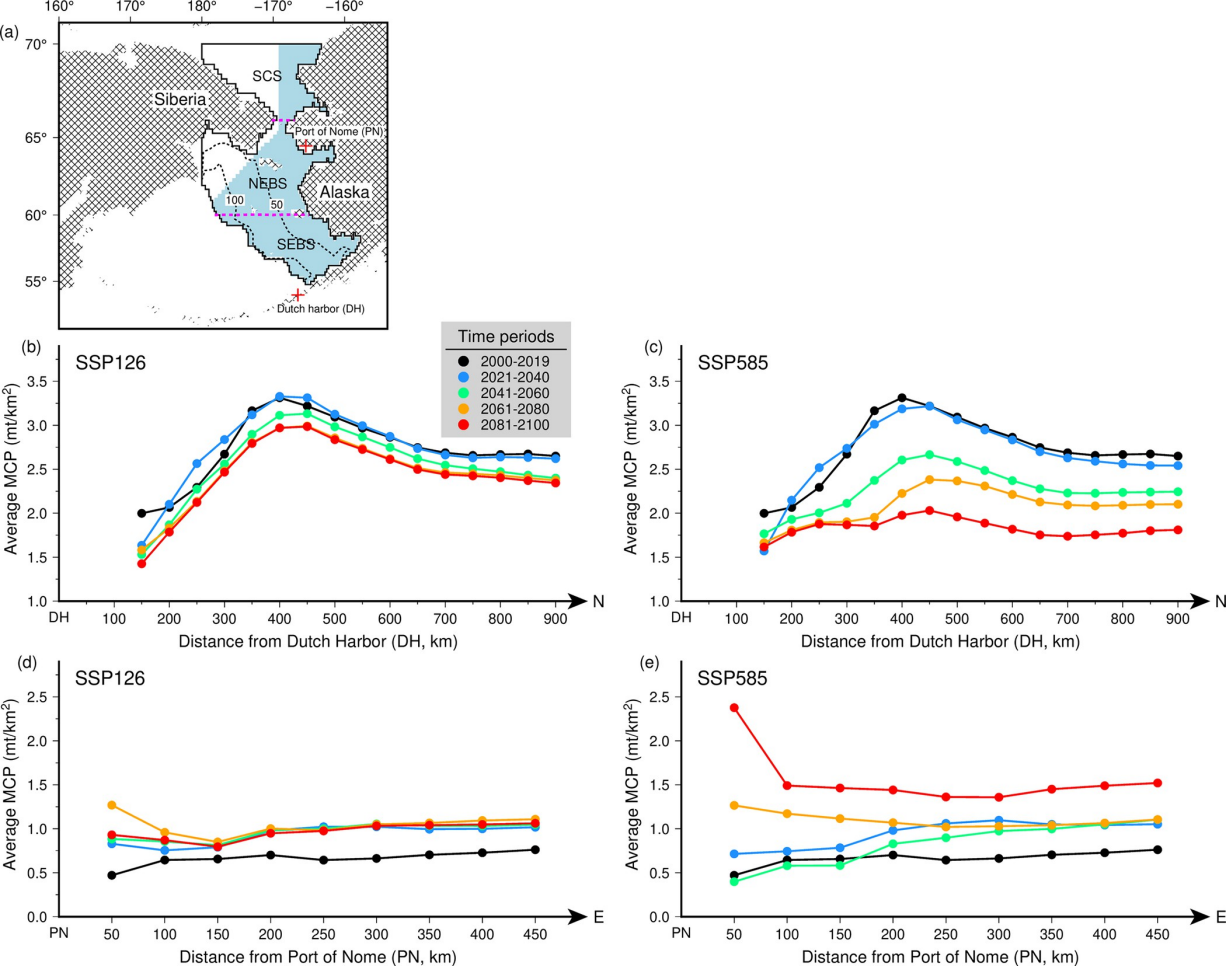
(a) Locations of the major and northern fishing ports (red crosses) in the Eastern Bering Sea and averaged MCP within the US EEZ (light blue polygon) computed at each 100-km buffer zone from the (b-c) Dutch Harbor and (d-e) Port of Nome for the present (2000–2019) and future periods (2021–2100) under SSP126 and SSP585. Bathymetric contours (black broken lines) and latitudinal (pink dashed lines) domains of the study area are shown in (a).

The contrasts between the averaged MCP relative to the major landing ports in the EBS (Fig 5A), showed differences in abundance trends with distance away from the ports (Fig 5B–5E). Differences in MCP between these ports were also captured under the rest of the SSPs (S10 Fig in S1 File). In Dutch harbor, MCPs under SSP126 (Fig 5B) showed minimal changes relative to SSP585 (Fig 5C). Under the latter, MCP peaks were observed at 400 km away from the port, with significant declines in magnitude between 2041 and 2100. In contrast, in the Port of Nome, future MCPs were generally higher than the present-day under SSP126 (Fig 5D) and SSP585 (Fig 5E). High MCPs were also observed at distances adjacent to the port.

**Fig 6:**
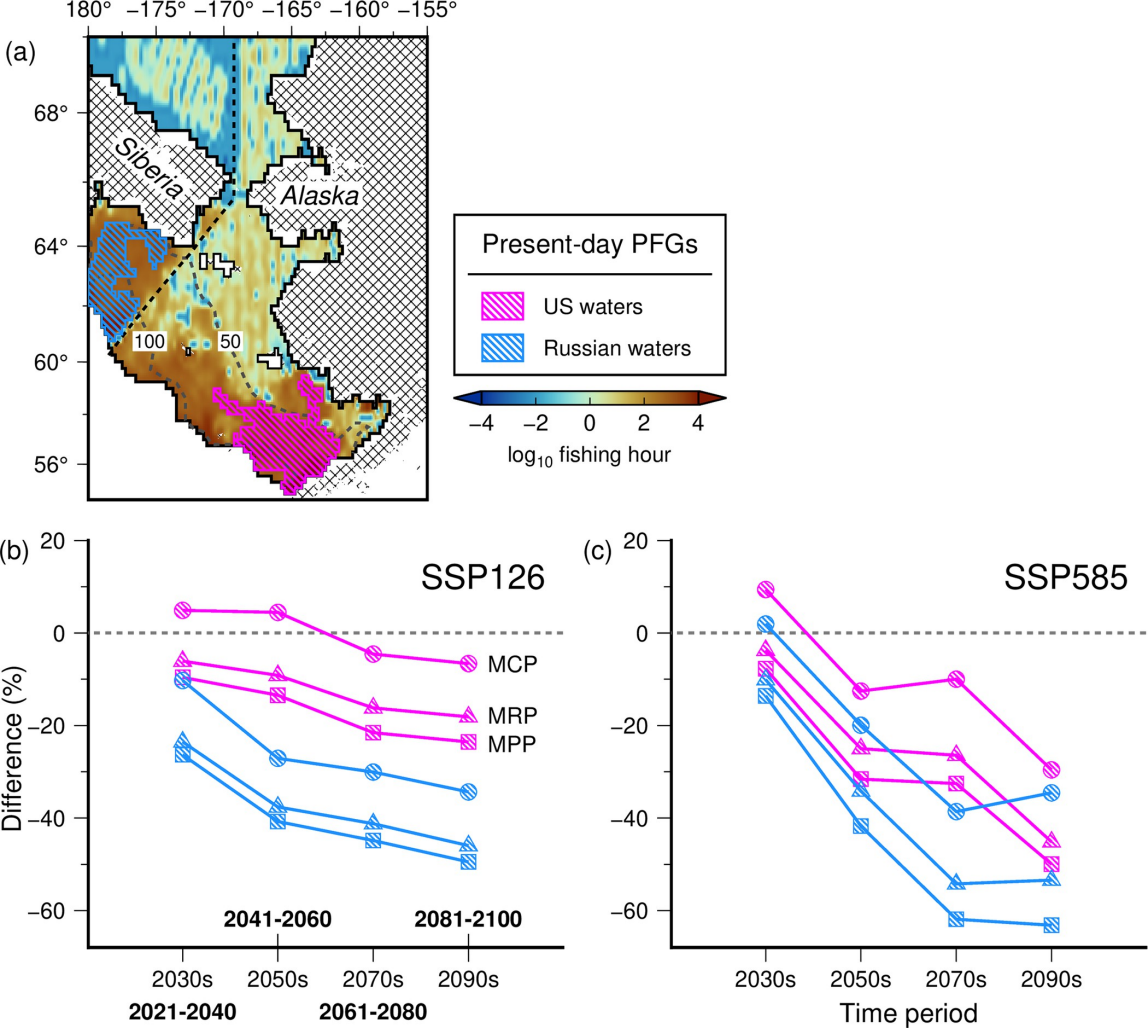
(a) Spatial distribution of aggregated fishing effort from 2012–2020, overlain with present-day fishing grounds (fishing hour ≥ 1000 hrs). Bottom panels showed the differences between present (2000–2019) and future (2021–2100) maximum catch (circles), revenue (triangles), and profit (squares) potential for all species under (b) SSP126 and (c) SSP585 in the US (magenta shapes and lines) and Russian (blue shapes and lines) fishing grounds, respectively.

Further, the maximum catch, revenue, and profit potential for these species between the different present-day fishing grounds in the US and Russian waters (Fig 6A) have also revealed distinct contrast between the low (SSP126) and high (SSP585) future climate scenarios (Fig 6B and 6C), with significant and steep declines in the MRPs and MPPs in the latter. Moreover, slight increases in MCP in response to shifting species distributions in the US fishing ground during 2021–2040 and 2041–2060 under contrasting climate scenarios did not translate to increases in the MRP and MPP. Between these fishing grounds, Russian waters further exhibited higher declines in MCPs, MRPs, and MPPs relative to the US fishing ground under SSP126 (Fig 6B) and SSP585 (Fig 6C). These contrasting bioeconomic trends and patterns were also observed for the intermediate climate scenarios (SSP245 and SSP370; S11 Fig in S1 File).

## Discussion

Projected changes in the global biomass of marine fauna are negatively related to the warming levels, although, climate change and its impacts on species and fisheries vary largely between regions and communities [66–68]. In the EBS and Chukchi Sea, future projected changes in the biophysical variables highlighted the contrasting levels of changes in sea surface and bottom temperatures, sea ice concentration, and primary production under future SSPs. In particular, an earlier effort of modeling fish abundance in the EBS using CMIP5 climate scenarios showed comparable spatial patterns for present and future predictions (seven taxa, Greenland halibut was excluded in the modeled species pool) with the results from our current work [69]. In the Greenland, Norwegian, and Barents seas, poleward expansions of fish stocks (e.g., Atlantic cod, Haddock, and Greenland halibut) have also been projected to occur under future warming Arctic [70]. This concordance in model predictions across geographic regions and commercial species underpins the relevance of curbing future greenhouse gas emissions and maintaining sustainable shared socioeconomic pathways to abate climate change impacts on fish resources. Indeed, our results using updated CMIP6 projections of climate and productivity showed that fluctuations in the abundance of our target species increased with the degree of future climate change. Nonetheless, the magnitude of abundance changes varied across species, identifying potential winners and losers under climate change and hinting on the potential restructuring of future marine communities in the Pacific Arctic region. Overall, our findings support mounting evidence of climate change impacts on species distributions and abundance with intensities proportional to the level of greenhouse emissions [18, 66, 67, 71].

The implementation of simple bioeconomic analyses to spatio-temporal projections of species abundance provide pertinent insight into potential biological and economic impacts of future climate change on major commercial fisheries in the area. Projected increases in MCP in the NEBS and Chukchi Sea under the extreme warming and sea ice loss scenario in 2081–2100, did not result into significant increases in MRP and MPP. These findings suggest that future changes in the catch composition can alter the potential economic benefits from fisheries. Previous work showed that despite anticipated increases in biodiversity and abundance in high-latitude environments in response to global warming [1, 18], the economic value of shifting marine resources largely influences the present and future revenue and profit potential from fisheries [2, 62].

Comparison of the future averaged MCPs with distance away from major fishing and Arctic ports in the EBS under contrasting shared socioeconomic pathways, revealed divergent patterns relative to the present. These changes are likely to impact the future fishing fleet mobility in response to shifting fish abundance, leading to potential losses and gains in port landings of target species [72]. It also raises the need for fishing industry to consider the trade-offs of longer transit times between fishing grounds and ports with processing facilities [73]. It is worth noting that our analyses used fishing mortality rates that are at healthy levels, stressing the importance of fisheries management in adapting to and mitigating the negative impacts of climate change [27, 58]. Nonetheless, even for well-managed ecosystems such as the EBS, a high greenhouse gas emission scenario could lead to serious economic impacts on fisheries, potentially from the loss of high-value taxa. Notably, even at the present-day magnitudes of warming and sea ice loss, we have already witnessed instances of population shifts and declines for many lucrative fisheries [13, 74, 75]. With projected MCP increases in NEBS and Chukchi Sea, future MRPs close to the major Arctic port showed potential increases above present levels. With anticipated increase in species abundance in the region, northern ports may become increasingly important for local fishing communities.

Our analyses also highlight the future significance of shifting marine fisheries resources between the US and Russian EEZ. This raises potential issues for governance and management of transboundary fish stocks and elevated risks of fisheries conflicts under continuing climatic changes [76–78]. For instance, the movement of fish stocks/populations out of their traditional fishing grounds can be challenging for resource managers and stakeholders [79], exacerbating the risks of overexploitation and the race-to-fish (i.e., competitive harvesting) of transboundary fishery resources [80]. Further, potential declines and/or extirpation of cold-adapted species of high economic value at their current distributional ranges may also lead to a potential switch to low value commercially-targeted species. The projected decreases in abundances of walleye pollock and Pacific cod within the US EEZ are likely to affect their commercial landings as their abundance shifts towards the Russian EEZ of the NEBS and Chukchi Sea.

The close proximity of COGs of abundance of these target species under future climate conditions also points to enhanced geographical overlap in species distributional ranges. From an ecological perspective, this could lead to elevated resource and space competition, increased predation pressures, density-dependent population changes, overfishing of less productive taxa and increased by-catch of non-target species with increasing fishing ground overlaps. In the EBS, recent declines in winter sea ice and summer cold pool extent led to increased predation intensity for juvenile walleye pollock by groundfish predators (e.g., arrowtooth flounder and Pacific halibut) [81]. Under future declines of winter sea ice and the disappearance of the cold pool feature in the EBS, high predation pressure on pelagic taxa due to the increased number of groundfish predators could be pervasive. Likewise, incidental catches of prohibited by-catch species from trawling gear (e.g., salmon and halibut) in the EBS posed conservation concerns as their biomass and population declines have been common for decades [82]. By-catch avoidance could be more challenging in the future with anticipated changes in the habitat overlap across our target species. Poleward shifts in the abundance and distribution of warm-adapted taxa are also disruptive to the ecological stability of Arctic marine ecosystems through potential reorganization of marine communities (e.g., borealization) and concurrent changes in collective interplay of multi-species interactions, influencing the ecosystems’ vulnerability and resilience to climate change [19, 83, 84]. We recognize, though, that our abundance projections are only taxon-specific, and as such multi-species interactions could lead to less future overlap in COGs than anticipated by single-species models.

Our scenario-based projections of climate changes in the potential availability of major commercially-exploited fisheries in the EBS and Chukchi Sea provided an outlook into future climate change impacts and could be useful for devising climate-ready adaptation and conservation measures for mitigation. We recognize that our model projections and scenario-based exercises are subject to sources of uncertainties from our simple biological and economic assumptions, and the limited subset of commercial fisheries included in our analyses. In particular, abundances predicted by machine learning models were taken to represent species-specific biomass used for deriving biological estimates of fishable biomass. Although we recognize that this approach likely does not entirely represent the actual standing stock biomass. Fish biomass estimates used in stock assessments are derived from population dynamics fishery models [85] that requires large amount of population-level information not available for the current analyses. Additionally, to compute for the biological estimates for the present-day and future periods, we used published species-specific biological parameters for the US BSAI species stocks [58]. These biological parameters were assumed to remain constant across future time periods with subsequent changes in maximum catch, revenue, and profit potential being solely driven by projected species abundance changes under future climate and shared socioeconomic pathways. We note, however, that this is a common practice in literature given the difficulty in projecting these biological parameters into the future [12, 27, 58].

Secondly, our economic analysis is intended only as a simple first-cut attempt to elucidate potential impacts of climate-driven changes in species abundance, potential catch, revenue, and profit. While we used the best data currently available for our study, there are several limitations and assumptions that should be considered when interpreting our results. For instance, the economic parameters (e.g. ex-vessel prices and fishing costs) were based on published values representing regional prices and costs across fishing gear types [12, 58] and were assumed to remain constant over time. However, these economic parameters are highly dynamic and are expected to change with consumer behavior, market preferences, and fisheries subsidies [12].

Another source of uncertainty in interpreting model projections of species distributions and abundance are the inherent structural uncertainties from outputs of Earth System Models discussed in detail from previous work [66, 86]. Notably, persistent regional uncertainties in projections of ocean net primary production (NPP) of CMIP6 models remain a challenge for assessing impacts on ecosystem services, despite the reductions in inter-model variability since CMIP5 [87]. The substantial disagreement in the regional magnitude and distributions of NPP in CMIP6 stems from the incomplete understanding and constraints from the lack of sufficient observation data (e.g., large uncertainties in the representation of biogeochemical processes and ocean physics) [87]. Future research can focus on improving the regional model skill of global climate models by integrating updated observation data (e.g., ship-based oceanographic surveys and monitoring) into ESMs [87, 88].

Despite these limitations, our results showed that for well-managed marine ecosystems such as the EBS, effects of climate change can, to some extent, have limited economic impact in the near-term (2021–2040) under the low to moderate levels of climatic changes. Nonetheless, as climate change approaches extreme conditions of warming and sea ice loss, mitigation and conservation efforts could demand changes of current conservation and management strategies to ameliorate the negative impacts on fisheries in the region. This is in agreement with earlier findings underscoring the benefits of ecosystem-based fisheries management in the EBS, dependent on the temporal scale and intensity of anticipated change [89]. Thus, the need for forward-looking and climate-smart solutions to manage, conserve, and rebuild dwindling and shifting marine fisheries resources are necessary to support future global food and nutritional security and sustain fisheries-reliant sectors under climate change [27, 28, 90].

## Supporting information

S1 File Supplemetary materials for analyses on future abundance projections under contrasting time periods and climate scenarios. (DOCX)
