# Correction: Future redistribution of fishery resources suggests biological and economic trade-offs according to the severity of the emission scenario

**DOI:** 10.1371/journal.pone.0321041

**Published:** 2025-03-25

**Authors:** Irene D. Alabia, Jorge García Molinos, Takafumi Hirata, Daiju Narita, Toru Hirawake

The second author’s initials appear incorrectly in the citation. The correct citation is: Alabia ID, García Molinos J, Hirata T, Narita D, Hirawake T (2024) Future redistribution of fishery resources suggests biological and economic trade-offs according to the severity of the emission scenario. PLoS ONE 19(6): e0304718. https://doi.org/10.1371/journal.pone.0304718
