# Supplementary material for: Future redistribution of fishery resources suggests biological and economic trade-offs according to the severity of the emission scenario
Source: PLoS One. 2024 Jun 6;19(6):e0304718. doi: 10.1371/journal.pone.0304718 (PMC11156307; doi:10.1371/journal.pone.0304718)
Supplement: S1 File — (DOCX) [file pone.0304718.s001.docx]

**Future redistribution of fishery resources suggests biological and economic trade-offs according to the severity of the emission scenario**

Irene D. Alabia^1*^, Jorge García Molinos^1^, Takafumi Hirata^1^,

Daiju Narita^2^, and Toru Hirawake^3^

^1^Arctic Research Center, Hokkaido University, N21 W11 Kita-ku, Sapporo, Hokkaido, Japan

^2^Graduate School and College of Arts and Sciences, The University of Tokyo, 3-8-1 Komaba, Meguro-ku, Tokyo, Japan

^3^National Institute of Polar Research / The Graduate University for Advanced Studies, SOKENDAI, 10-3, Midori-cho, Tachikawa, Tokyo, Japan

Correspondence: irenealabia@arc.hokudai.ac.jp

**Supplementary Materials**

**List of Tables**

**Table S1.** List of fish and crab species used for analyses and averaged landings for Alaska (in metric tons, mt) and market values (in million US dollars) in the last two decades (2000-2019). Values in parentheses are the percentages of landings for each species relative to the total averaged catch in Alaska between 2000 and 2019 (https://www.fisheries.noaa.gov/foss/).

**Table S2.** Optimal hyperparameters based on the minimum root mean square error (RMSE) values (model runs = 2700) and the explained variance of species-specific random forest (RF) abundance models.

**Table S3.** Optimal hyperparameters based on the minimum root mean square error (RMSE) values (model runs = 2700) and the explained variance of species-specific boosted regression trees (BRT) abundance models.

**Table S4.** Biological reference and economic parameters extracted from Gaines et al. [1] for the eight species used in this study. Historical reference values were based on the RAM legacy database version 2.95 [2,3]. Ex-vessel prices (p) were based on FAO export data and published estimates of average ex-vessel values [2]. The reference values were standardized to a common baseline year (2012).

**Table S5.** Basin-averaged future (2021-2100) percentage of maximum profit potential (MPP) for all species relative to present (2000-2019) under the different climate, prices and costs scenarios.

**Table S6.** Relative environmental variable importance based on permutation (*n* = 100) in the final random forest (RF) models. Values in red are the three most important environmental variables in modeling species-specific abundance.

**Table S7.** Relative environmental variable importance in final boosted regression trees (BRT) model. Values in red are the three most important environmental variables in modeling species-specific abundance.

**Table S8.** Basin-averaged future (2021-2100) percentage of abundance changes for each species relative to present (2000-2019) under the different climate scenarios.

**List of Figures**

**Figure S1.** (a) Spatial distribution of aggregated fishing effort from 2012-2020, overlain with present-day fishing grounds (fishing hour ≥ 1000 hrs). Bottom panels show differences between the present (2000-2019) and future (2021-2100; SSP585 scenario) maximum catch (circles), revenue (triangles), and profit (squares) potential under the different price and cost scenarios in the US (magenta shapes and lines) and Russian (blue shapes and lines) fishing grounds, respectively.

**Figure S2.** Map showing the continental shelf areas (depth ≤ 200m) of the Pacific Arctic Region (PAR), divided into the Southeastern Bering Sea (SEBS; 52-60°N) and Northeastern Bering Sea (NEBS;60-66°N), and Southern Chukchi Sea (SCS). Latitudinal boundaries of each region are shown in pink dashed lines. Overlain on the map are the major (Dutch harbor) and one of the northern commercial ports (Port of Nome) surrounding the PAR (red circles). The yellow line corresponds to the US-Russia exclusive economic zone (EEZ) boundary, with Eastern Bering Sea commercial fisheries groundfish district marked by diagonal lines. Topographical domains (inner, 0-50m; middle, 50-100m; and outer, 100-200m) are delineated by the isobaths (white lines).

**Figure S3.** Spatial distributions of (a) summer sea bottom temperature (SSBT), (b) winter sea bottom temperature (WSBT), (c) winter sea surface temperature (WSST), (d) winter sea ice concentration (WSIC), and (e) summer net primary production (SNPP) between present (2000-2019) and future periods (2021-2100) under the SSP126 scenario. Overlain on (a) is the cold pool feature (SSBT < 2°C; dashed lines) and bathymetric contours (solid gray lines).

**Figure S4.** Spatial distributions of (a) summer sea bottom temperature (SSBT), (b) winter sea bottom temperature (WSBT), (c) winter sea surface temperature (WSST), (d) winter sea ice concentration (WSIC), and (e) summer net primary production (SNPP) between present (2000-2019) and future periods (2021-2100) under the SSP245 scenario. Overlain on (a) is the cold pool feature (SSBT < 2°C; dashed lines) and bathymetric contours (solid gray lines).

**Figure S5.** Spatial distributions of (a) summer sea bottom temperature (SSBT), (b) winter sea bottom temperature (WSBT), (c) winter sea surface temperature (WSST), (d) winter sea ice concentration (WSIC), and (e) summer net primary production (SNPP) between present (2000-2019) and future periods (2021-2100) under the SSP370 scenario. Overlain on (a) is the cold pool feature (SSBT < 2°C; dashed lines) and bathymetric contours (solid gray lines).

**Figure S6.** Abundance-weighted **c**enter of gravity ±1 standard deviation (gray lines) of the species-specific abundance throughout the present-day fishing ground (pink diagonal patterns; fishing hours ≥ 1000 hours between 2012-2020) in the US waters for the (a) present-day and future periods (2021-2040, first panels; 2041-2060, second panels; 2061-2080 , third panels; 2081-2100, fourth panels) under the (b) SSP126, (c) SSP245, (d) SSP370, and (e) SSP585 socioeconomic pathways. Dashed lines represent the bathymetic contours.

**Figure S7.** Abundance-weighted center of gravity ±1 standard deviation (gray lines) of the species-specific abundance throughout the present-day fishing ground (pink diagonal lines; fishing hours ≥ 1000 hours between 2012-2020) in the Russian waters for the (a) present-day and future periods (2021-2040, first panels; 2041-2060, second panels; 2061-2080 , third panels; 2081-2100, fourth panels) under the (b) SSP126, (c) SSP245, (d) SSP370, and (e) SSP585 socioeconomic pathways.

**Figure S8.** Spatial distributions of modeled abundance for (a) present (2000-2019) and future (2021-2100) periods under (b) SSP126, (c) SSP245, (d) SSP370, and (e) SSP585 for all species classified based on their catch and economic values. Overlain are the US-Russian border (dashed lines) and bathymetric contours (white solid lines) in the Eastern Bering and Chukchi seas.

**Figure S9.** Predicted future (2021-2040; 2061-2080) percent changes in the maximum catch potential (MCP) under (a) SSP126, (b) SSP245, and (c) SSP370, and (d) SSP585 for eight major fisheries relative to the present (2000-2019). Overlain are the US-Russia EEZ boundary (dashed line) and bathymetric contours (solid gray lines).

**Figure S10.** (a) Locations of the major and northern fishing ports (red crosses) in the Eastern Bering Sea and averaged MCP within the US EEZ (light blue polygon) computed at each 100-km buffer zone from the (b-c) Dutch Harbor and (d-e) Port of Nome for the present (2000-2019) and future periods (2021-2100) under SSP245 and SSP370. Bathymetric contours (black broken lines) and latitudinal (pink dashed lines) domains of the study area are shown in (a).

**Figure S11.** (a) Spatial distribution of aggregated fishing effort from 2012-2020, overlain with present-day potential fishing grounds (PFGs, fishing hour ≥ 1000 hrs). Differences between present (2000-2019) and future (2021-2100) maximum catch (circles), revenue (triangles), and profit (squares) potential for all species under (b) SSP245 and (c) SSP370 within the US (magenta shapes and lines) and Russian (blue shapes and lines) fishing grounds, respectively.

**Supplementary Tables**

**Table S1.** List of fish and crab species used for analyses and averaged landings for Alaska (in metric tons, mt) and market values (in million US dollars) in the last two decades (2000-2019). Values in parentheses are the percentages of landings for each species relative to the total averaged catch in Alaska between 2000 and 2019 (https://www.fisheries.noaa.gov/foss/).

| **Scientific name** | **Common name** | **Alaska average (2000-2019)** | |
| --- | --- | --- | --- |
|  |  | **Landings (x10^3^ mt)** | **Value (x10^6^ USD)** |
| *Gadus chalcogrammus* | Walleye pollock | 1368.91 (57.28) | 337.19 |
| *Gadus macrocephalus* | Pacific cod | 262.08 (10.97) | 164.91 |
| *Limanda aspera* | Yellowfin sole | 109.74 (4.59) | 76.21 |
| *Atheresthes stomias* | Arrowtooth flounder | 27.43 (1.15) | 34.64 |
| *Chionoecetes opilio* | Snow crab | 18.95 (0.79) | 10.45 |
| *Hippoglossoides elassodon* | Flathead sole | 15.59 (0.65) | 5.20 |
| *Pleuronectes quadrituberculatus* | Alaska plaice | 12.36 (0.52) | 4.54 |
| *Reinhardtius hippoglossoides* | Greenland halibut | 2.81 (0.12) | 1.91 |

**Table S2.** Optimal hyperparameters based on the minimum root mean square error (RMSE) values (model runs = 2700) and the explained variance of species-specific random forest (RF) abundance models.

| **Species** | **Observations (*n*)** | **Number of features to split at each node (mtry)** | **Minimum**  **node size** | **Number of trees**  **(n.trees)** | **RMSE** | **Deviance explained (%)** |
| --- | --- | --- | --- | --- | --- | --- |
| Walleye pollock | 8836 | 3 | 2 | 900 | 1.255 | 72.13 |
| Pacific cod | 8836 | 4 | 3 | 900 | 1.177 | 70.51 |
| Yellowfin sole | 8832 | 6 | 4 | 1000 | 1.241 | 84.59 |
| Arrowtooth flounder | 8851 | 2 | 3 | 1000 | 1.269 | 80.89 |
| Snow crab | 8836 | 3 | 6 | 800 | 1.406 | 71.48 |
| Flathead sole | 8836 | 5 | 7 | 900 | 1.542 | 68.86 |
| Alaska plaice | 8835 | 4 | 6 | 1000 | 1.578 | 68.81 |
| Greenland halibut | 8837 | 4 | 2 | 600 | 1.270 | 55.24 |

**Table S3.** Optimal hyperparameters based on the minimum root mean square error (RMSE) values (model runs = 2700) and the explained variance of species-specific boosted regression trees (BRT) abundance models.

| **Species** | **Observations (*n*)** | **Shrinkage (learning rate)** | **Interaction depth** | **Number of trees (n.trees)** | **RMSE** | **Deviance explained (%)** |
| --- | --- | --- | --- | --- | --- | --- |
| Walleye pollock | 8836 | 0.03 | 10 | 77 | 1.614 | 71.60 |
| Pacific cod | 8836 | 0.04 | 10 | 75 | 1.421 | 70.95 |
| Yellowfin sole | 8832 | 0.03 | 10 | 79 | 1.746 | 86.92 |
| Arrowtooth flounder | 8851 | 0.04 | 10 | 60 | 1.444 | 80.48 |
| Snow crab | 8836 | 0.05 | 10 | 49 | 1.868 | 71.60 |
| Flathead sole | 8836 | 0.05 | 9 | 67 | 1.531 | 70.12 |
| Alaska plaice | 8835 | 0.05 | 10 | 278 | 1.819 | 70.65 |
| Greenland halibut | 8837 | 0.04 | 4 | 84 | 1.392 | 55.91 |

**Table S4.** Biological reference and economic parameters extracted from Gaines et al. [[1](#_ENREF_1)] for the eight species used in this study. Historical reference values were based on the RAM legacy database version 2.95 [[2](#_ENREF_2),[3](#_ENREF_3)]. Ex-vessel prices (p) were based on FAO export data and published estimates of average ex-vessel values [2]. The reference values were standardized to a common baseline year (2012).

| **Data source** | **Common name and species stock** | **b (B/B_MSY_)** | **U_MSY_** | **f (F/F_MSY_)** | **g (MSY/B_MSY_)** | **p (USD/mt)** |
| --- | --- | --- | --- | --- | --- | --- |
| AFSC-WPOLLEBS-1964-2013-CHING | Walleye pollock Eastern Bering Sea | 1.08 | 0.543 | 0.719 | 0.377 | 875.14 |
| AFSC-PCODBSAI-1964-2013-CHING | Pacific cod Bering Sea and Aleutian Islands | 1.18 | 0.340 | 0.774 | 0.443 | 2707.56 |
| AFSC-YSOLEBSAI-1949-2010-STACHURA | Yellowfin sole Bering Sea and Aleutian Islands | 1.81 | 0.130 | 0.615 | 0.087 | 966.52 |
| AFSC-ARFLOUNDBSAI-1970-2010-STACHURA | Arrowtooth flounder Bering Sea and Aleutian Islands | 2.50 | 0.290 | 0.090 | 0.176 | 2418.45 |
| AFSC-SNOWCRABBS-1978-2013-HIVELY | Snow crab Bering Sea | 1.10 | 1.580 | 0.373 | 0.275 | 4469.18 |
| AFSC-FLSOLEBSAI-1974-2010-STACHURA | Flathead sole Bering Sea and Aleutian Islands | 2.04 | 0.342 | 0.166 | 0.195 | 1339.12 |
| AFSC-ALPLAICBSAI-1972-2010-STACHURA | Alaska plaice Bering Sea and Aleutian Islands | 1.69 | 0.190 | 0.177 | 0.112 | 1608.81 |
| AFSC-GHALBSAI-1960-2009-STANTON | Greenland halibut Bering Sea and Aleutian Islands | 1.46 | 0.566 | 0.093 | 0.304 | 4075.50 |

**Table S5.** Basin-averaged future (2021-2100) percentage of maximum profit potential (MPP) for all species relative to present (2000-2019) under the different climate, prices and costs scenarios.

| **Climate Scenario** | **SSP126** | | | | **SSP245** | | | |
| --- | --- | --- | --- | --- | --- | --- | --- | --- |
| **Price & Cost Scenario/ Period** | **2021-2040** | **2041-2060** | **2061-2080** | **2081-2100** | **2021-2040** | **2041-2060** | **2061-2080** | **2081-2100** |
| Original Prices & Costs | -15.72 | -22.74 | -26.21 | -28.25 | -9.95 | -23.30 | -30.77 | -40.55 |
| Increasing Costs | -16.36 | -24.04 | -28.24 | -30.87 | -10.60 | -24.75 | -32.99 | -43.55 |
| Increasing Prices | -10.86 | -13.72 | -13.12 | -11.27 | -4.80 | -14.18 | -18.17 | -25.66 |
| Increasing Prices & Costs | -11.50 | -15.02 | -15.14 | -13.90 | -5.45 | -15.63 | -20.39 | -28.66 |
|  |  |  |  |  |  |  |  |  |
| **Climate Scenario** | **SSP370** | | | | **SSP585** | | | |
| **Price & Cost Scenario/ Period** | **2021-2040** | **2041-2060** | **2061-2080** | **2081-2100** | **2021-2040** | **2041-2060** | **2061-2080** | **2081-2100** |
| Original Prices & Costs | -10.38 | -23.46 | -37.66 | -44.28 | -13.22 | -31.66 | -42.68 | -46.83 |
| Increasing Costs | -11.05 | -24.99 | -40.06 | -47.38 | -13.89 | -33.22 | -45.04 | -50.03 |
| Increasing Prices | -5.23 | -14.27 | -25.92 | -30.04 | -8.22 | -23.27 | -31.71 | -33.01 |
| Increasing Prices & Costs | -5.90 | -15.80 | -28.31 | -33.14 | -8.89 | -24.83 | -34.08 | -36.20 |

**Table S6.** Relative environmental variable importance based on permutation (*n* = 100) in the final random forest (RF) models. Values in red are the three most important environmental variables in modeling species-specific abundance.

| Species | WSST | WSIC | WSBT | SSBT | SNPP | Depth |
| --- | --- | --- | --- | --- | --- | --- |
| Walleye pollock | 1.38 | 1.06 | 1.92 | 0.84 | 1.31 | 6.35 |
| Pacific cod | 0.93 | 1.15 | 1.72 | 1.07 | 0.92 | 4.40 |
| Yellowfin sole | 1.28 | 1.09 | 2.74 | 1.44 | 1.44 | 13.08 |
| Arrowtooth flounder | 2.33 | 1.59 | 3.86 | 2.09 | 1.41 | 3.01 |
| Snow crab | 1.53 | 0.68 | 1.86 | 2.12 | 3.53 | 3.98 |
| Flathead sole | 1.76 | 1.32 | 3.23 | 1.17 | 1.20 | 6.07 |
| Alaska plaice | 1.21 | 0.86 | 4.33 | 1.32 | 1.59 | 7.16 |
| Greenland halibut | 1.01 | 0.59 | 1.49 | 1.85 | 0.57 | 2.58 |

**Table S7.** Relative environmental variable importance in the final boosted regression trees (BRT) model. Values in red are the three most important environmental variables in modeling species-specific abundance.

| Species | WSST | WSIC | WSBT | SSBT | SNPP | Depth |
| --- | --- | --- | --- | --- | --- | --- |
| Walleye pollock | 2.77 | 1.13 | 13.28 | 2.38 | 4.25 | 76.19 |
| Pacific cod | 10.26 | 3.46 | 5.95 | 6.08 | 4.22 | 70.02 |
| Yellowfin sole | 1.73 | 1.17 | 1.16 | 2.68 | 7.06 | 86.20 |
| Arrowtooth flounder | 0.76 | 0.89 | 72.92 | 7.30 | 10.71 | 7.42 |
| Snow crab | 3.52 | 0.57 | 1.93 | 18.60 | 34.76 | 40.61 |
| Flathead sole | 7.33 | 3.62 | 32.99 | 3.95 | 3.55 | 48.55 |
| Alaska plaice | 3.74 | 3.36 | 8.89 | 5.96 | 9.28 | 68.77 |
| Greenland halibut | 6.66 | 4.65 | 5.92 | 32.38 | 8.40 | 42.00 |

**Table S8.** Basin-averaged future (2021-2100) percentage of abundance changes for each species relative to present (2000-2019) under the different climate scenarios.

| **Climate Scenario** | **SSP126** | | | | **SSP245** | | | |
| --- | --- | --- | --- | --- | --- | --- | --- | --- |
| **Species/Period** | 2021-2040 | 2041-2060 | 2061-2080 | 2081-2100 | 2021-2040 | 2041-2060 | 2061-2080 | 2081-2100 |
| Walleye pollock | 6.90 | -4.13 | -6.20 | -7.66 | 10.43 | 0.06 | -3.69 | -7.17 |
| Pacific cod | 13.65 | 13.95 | 18.98 | 9.25 | 18.96 | 31.23 | 30.84 | 19.88 |
| Yellowfin sole | 24.60 | 28.67 | 35.86 | 36.13 | 14.62 | 34.69 | 45.07 | 60.42 |
| Arrowtooth flounder | 41.28 | 61.43 | 70.44 | 70.67 | 50.96 | 96.79 | 95.03 | 105.48 |
| Snow crab | -38.81 | -47.88 | -55.64 | -56.88 | -29.69 | -55.50 | -69.16 | -85.78 |
| Flathead sole | 33.56 | 40.91 | 55.96 | 56.22 | 33.54 | 64.56 | 84.53 | 109.48 |
| Alaska plaice | 5.44 | 6.14 | 8.08 | 5.75 | -2.67 | -6.50 | -1.55 | -7.56 |
| Greenland halibut | -75.95 | -89.81 | -94.35 | -97.20 | -66.00 | -97.09 | -99.51 | -99.93 |
| **Total Abundance change** | 1.92 | -4.55 | -5.27 | -7.10 | 4.68 | -0.86 | -3.65 | -7.09 |
|  |  |  |  |  |  |  |  |  |
| **Climate Scenario** | **SSP370** | | | | **SSP585** | | | |
| **Species/Period** | 2021-2040 | 2041-2060 | 2061-2080 | 2081-2100 | 2021-2040 | 2041-2060 | 2061-2080 | 2081-2100 |
| Walleye pollock | 11.48 | 7.44 | 0.31 | -1.45 | 8.74 | 0.02 | 1.80 | -6.00 |
| Pacific cod | 24.69 | 41.18 | 25.98 | 5.25 | 23.42 | 31.40 | 15.23 | -8.86 |
| Yellowfin sole | 16.40 | 33.31 | 48.69 | 65.81 | 22.39 | 43.01 | 60.46 | 110.40 |
| Arrowtooth flounder | 56.54 | 108.10 | 159.23 | 155.99 | 56.87 | 144.69 | 149.86 | 183.64 |
| Snow crab | -32.37 | -60.99 | -84.58 | -93.64 | -36.83 | -73.16 | -93.20 | -98.73 |
| Flathead sole | 40.42 | 73.11 | 120.36 | 129.02 | 43.01 | 96.92 | 125.10 | 168.12 |
| Alaska plaice | -4.88 | -13.14 | -27.61 | -38.48 | -4.93 | -20.88 | -37.33 | -70.58 |
| Greenland halibut | -72.14 | -98.89 | -99.96 | -99.98 | -70.31 | -99.79 | -99.99 | -100.00 |
| **Total abundance change** | 5.58 | 2.80 | -3.22 | -5.65 | 3.98 | -2.04 | -3.89 | -4.22 |

**Supplementary figures**


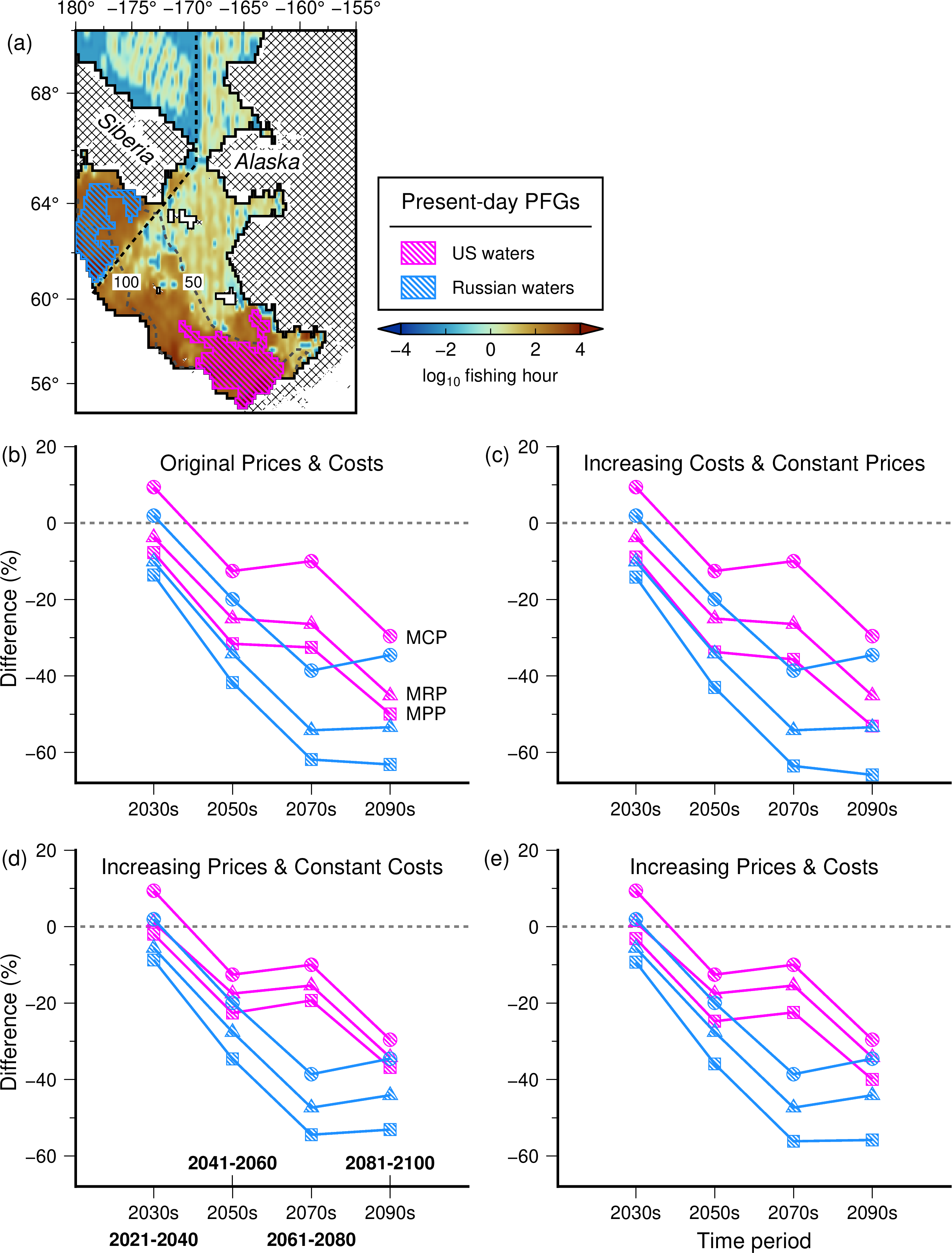


**Figure S1**. (a) Spatial distribution of aggregated fishing effort from 2012-2020, overlain with present-day fishing grounds (fishing hour ≥ 1000 hrs). Bottom panels show differences between the present (2000-2019) and future (2021-2100; SSP585 scenario) maximum catch (circles), revenue (triangles), and profit (squares) potential under the different price and cost scenarios in the US (magenta shapes and lines) and Russian (blue shapes and lines) fishing grounds, respectively.

**
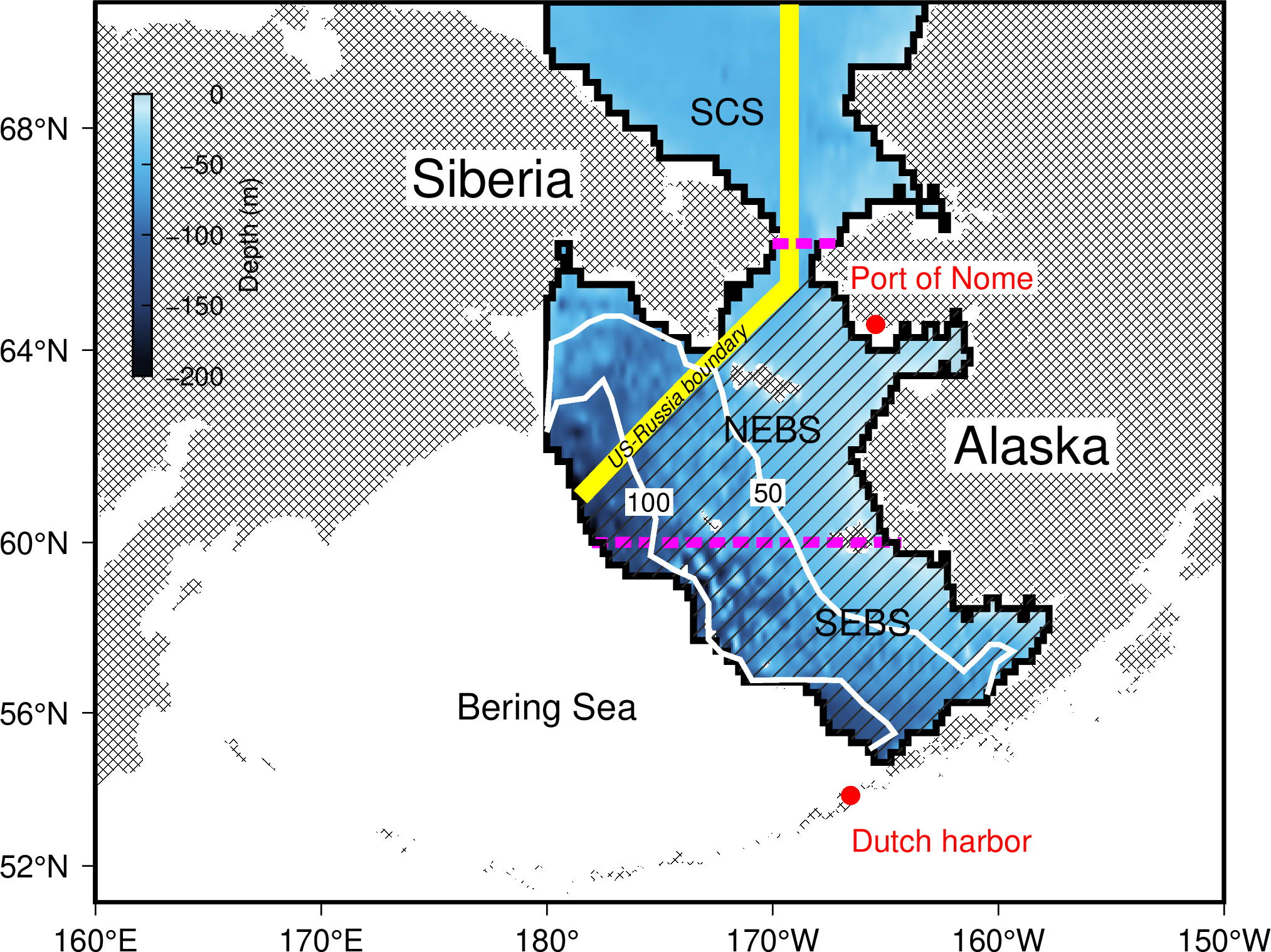
**

**Figure S2.** Map showing the continental shelf areas (depth ≤ 200m) of the Pacific Arctic Region (PAR), divided into the Southeastern Bering Sea (SEBS; 52-60°N) and Northeastern Bering Sea (NEBS;60-66°N), and Southern Chukchi Sea (SCS). Latitudinal boundaries of each region are shown in pink dashed lines. Overlain on the map are the major (Dutch harbor) and one of the northern commercial ports (Port of Nome) surrounding the PAR (red circles). The yellow line corresponds to the US-Russia exclusive economic zone (EEZ) boundary, with Eastern Bering Sea commercial fisheries groundfish district marked by diagonal lines. Topographical domains (inner, 0-50m; middle, 50-100m; and outer, 100-200m) are delineated by the isobaths (white lines).


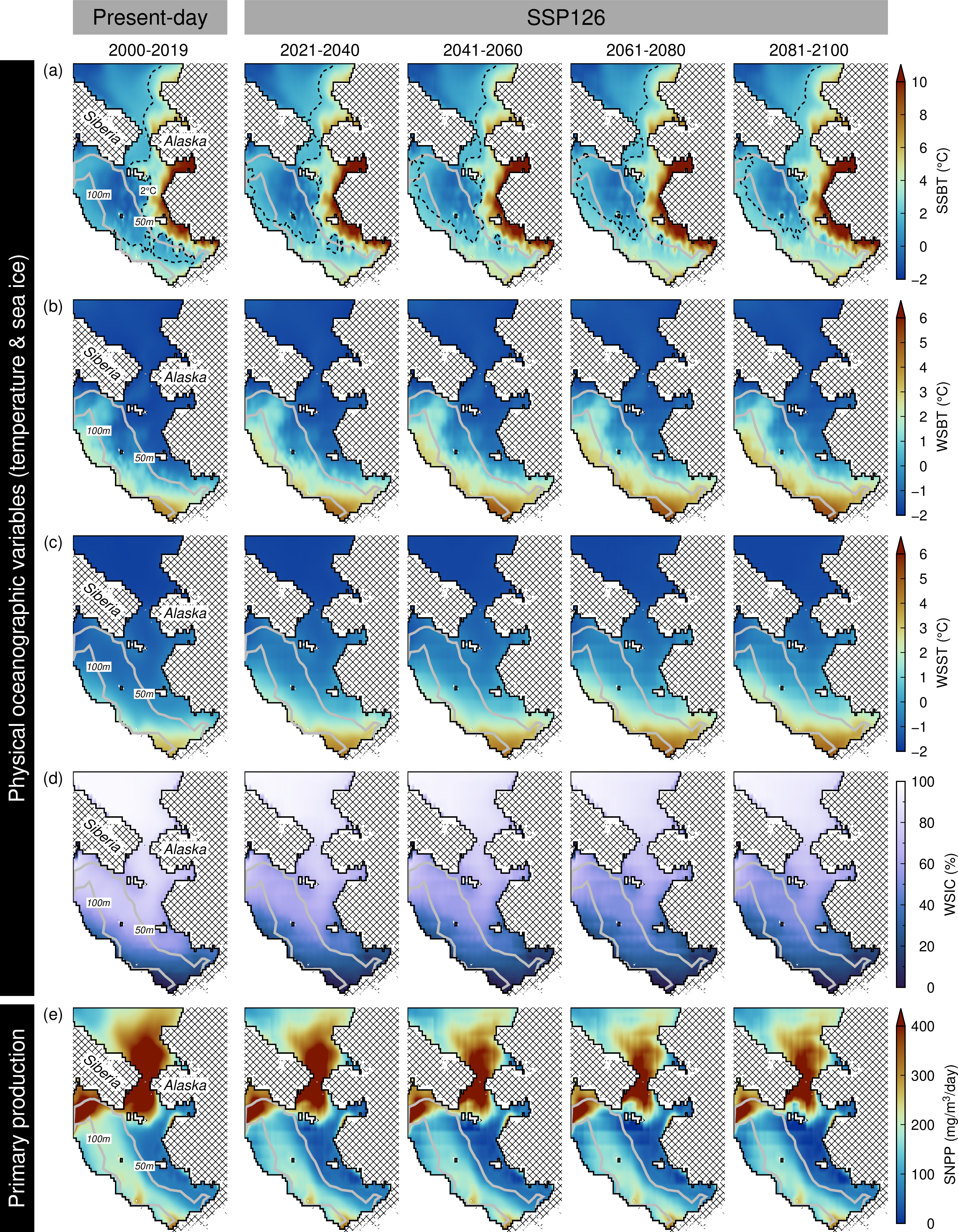


**Figure S3.** Spatial distributions of (a) summer sea bottom temperature (SSBT), (b) winter sea bottom temperature (WSBT), (c) winter sea surface temperature (WSST), (d) winter sea ice concentration (WSIC), and (e) summer net primary production (SNPP) between present (2000-2019) and future periods (2021-2100) under the SSP126 scenario. Overlain on (a) is the cold pool feature (SSBT < 2°C; dashed lines) and bathymetric contours (solid gray lines).


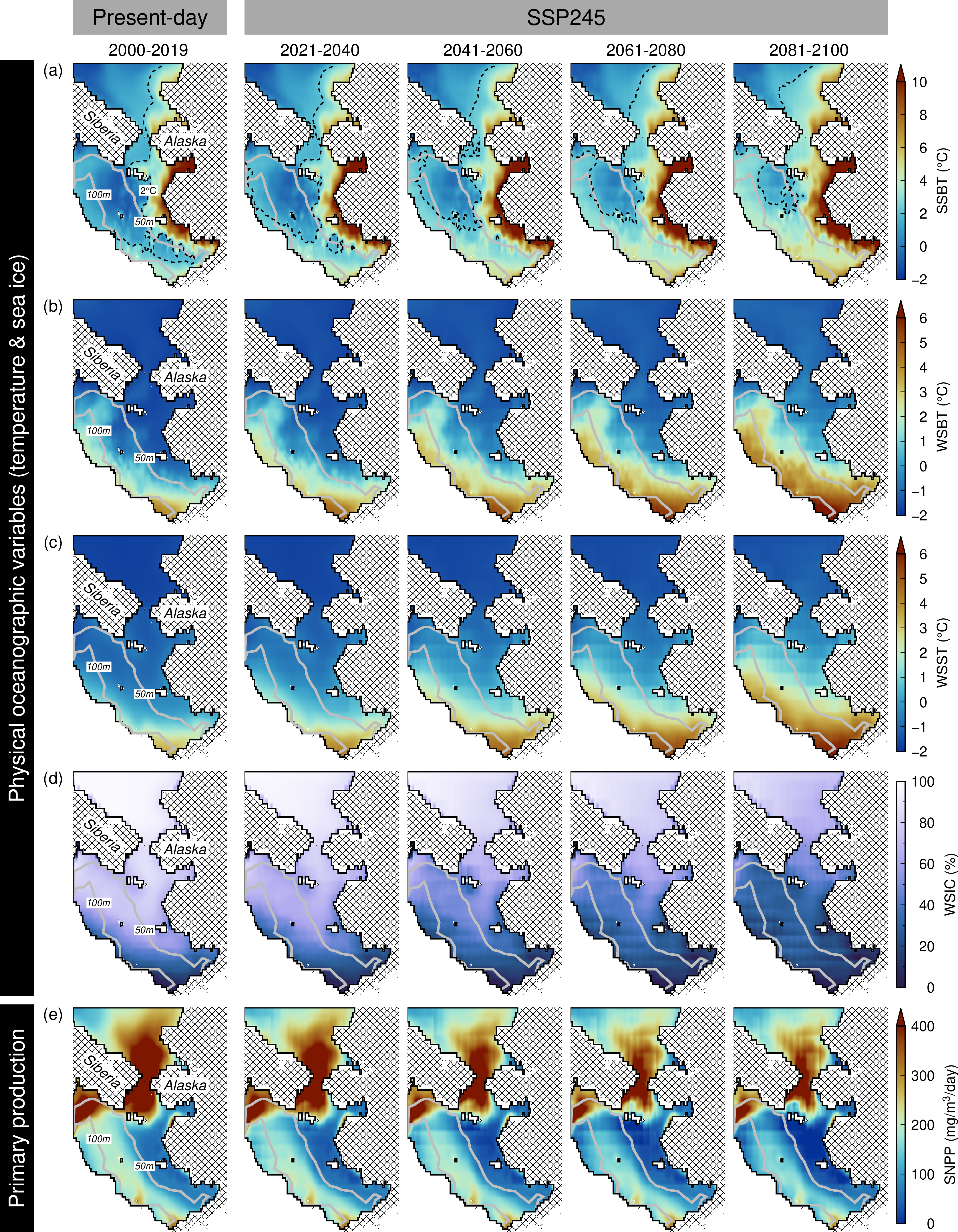


**Figure S4.** Spatial distributions of (a) summer sea bottom temperature (SSBT), (b) winter sea bottom temperature (WSBT), (c) winter sea surface temperature (WSST), (d) winter sea ice concentration (WSIC), and (e) summer net primary production (SNPP) between present (2000-2019) and future periods (2021-2100) under the SSP245 scenario. Overlain on (a) is the cold pool feature (SSBT < 2°C; dashed lines) and bathymetric contours (solid gray lines).


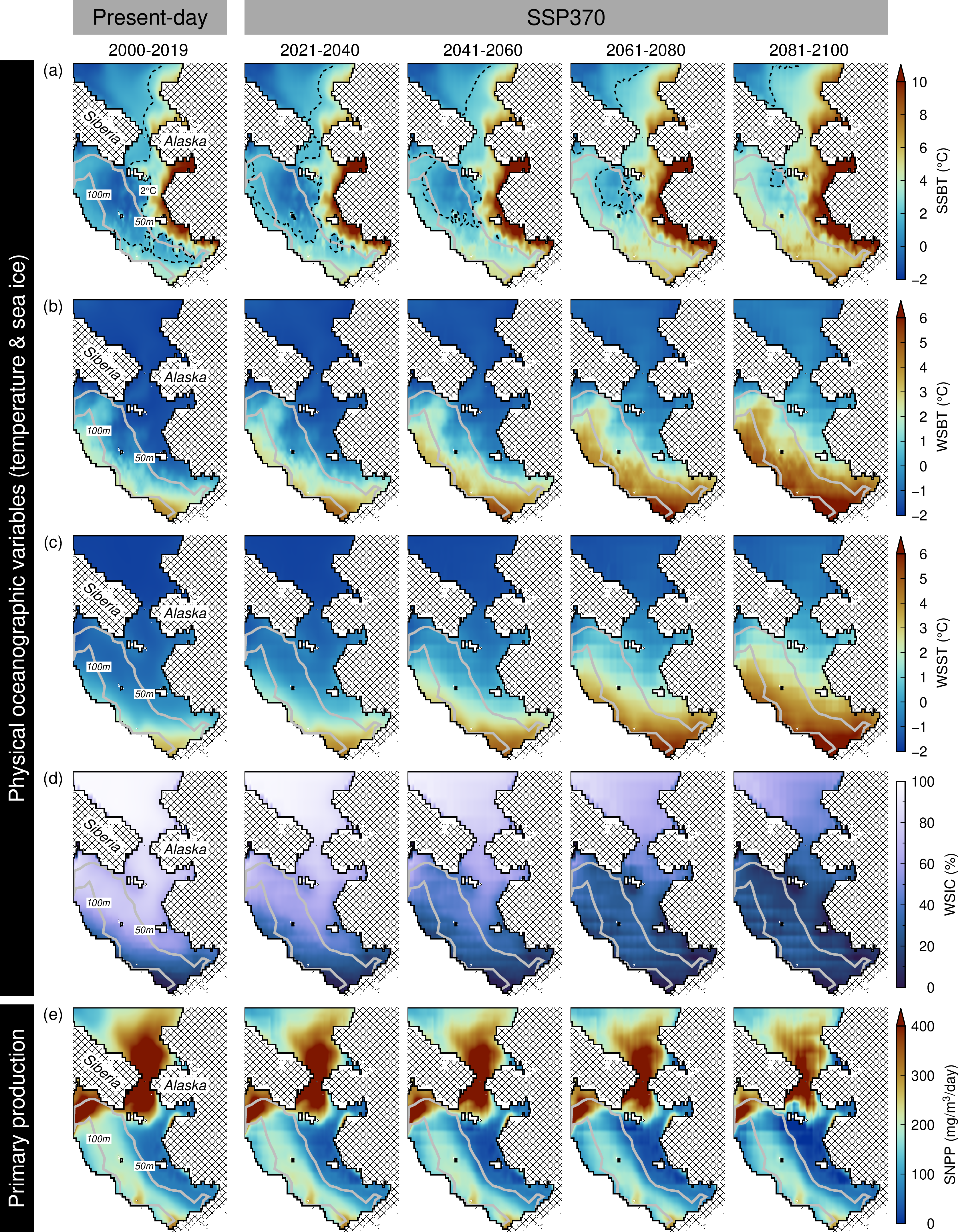


**Figure S5.** Spatial distributions of (a) summer sea bottom temperature (SSBT), (b) winter sea bottom temperature (WSBT), (c) winter sea surface temperature (WSST), (d) winter sea ice concentration (WSIC), and (e) summer net primary production (SNPP) between present (2000-2019) and future periods (2021-2100) under the SSP370 scenario. Overlain on (a) is the cold pool feature (SSBT < 2°C; dashed lines) and bathymetric contours (solid gray lines).


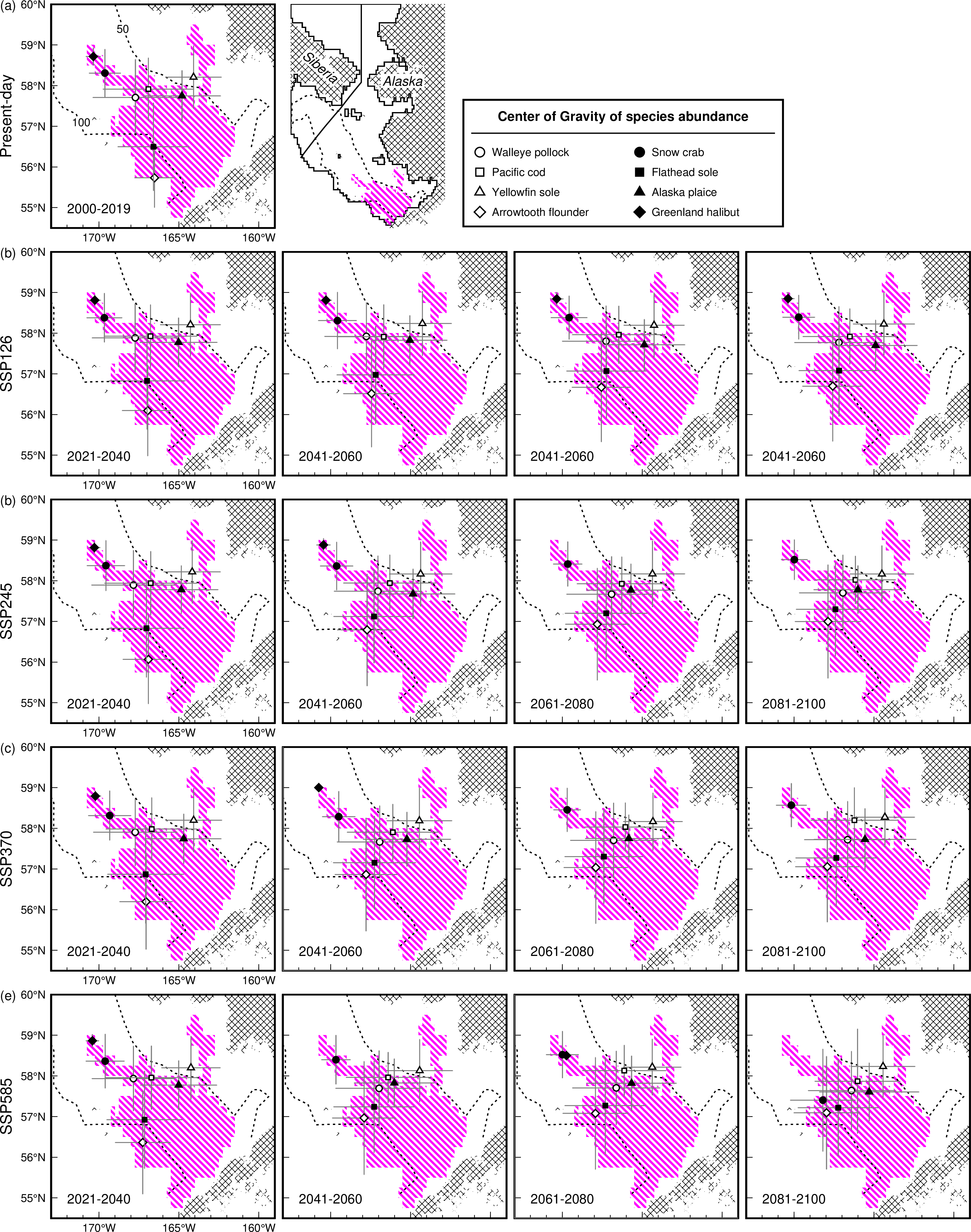


**Figure S6.** Abundance-weighted **c**enter of gravity ±1 standard deviation (gray lines) of the species-specific abundance throughout the present-day fishing ground (pink diagonal patterns; fishing hours ≥ 1000 hours between 2012-2020) in the US waters for the (a) present-day and future periods (2021-2040, first panels; 2041-2060, second panels; 2061-2080 , third panels; 2081-2100, fourth panels) under the (b) SSP126, (c) SSP245, (d) SSP370, and (e) SSP585 socioeconomic pathways. Dashed lines represent the bathymetic contours.

**
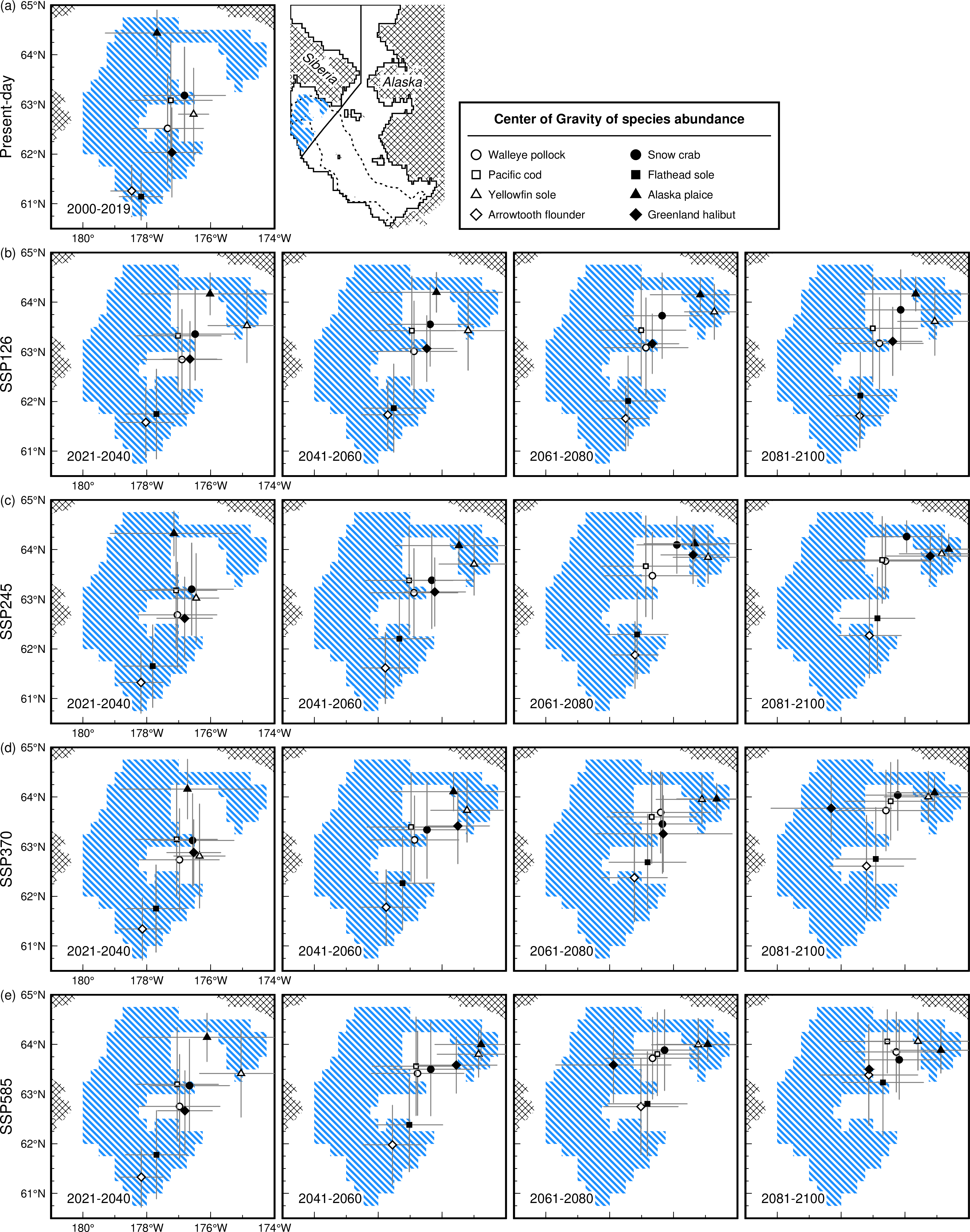
**

**Figure S7.** Abundance-weighted center of gravity ±1 standard deviation (gray lines) of the species-specific abundance throughout the present-day fishing ground (pink diagonal lines; fishing hours ≥ 1000 hours between 2012-2020) in the Russian waters for the (a) present-day and future periods (2021-2040, first panels; 2041-2060, second panels; 2061-2080 , third panels; 2081-2100, fourth panels) under the (b) SSP126, (c) SSP245, (d) SSP370, and (e) SSP585 socioeconomic pathways.

**
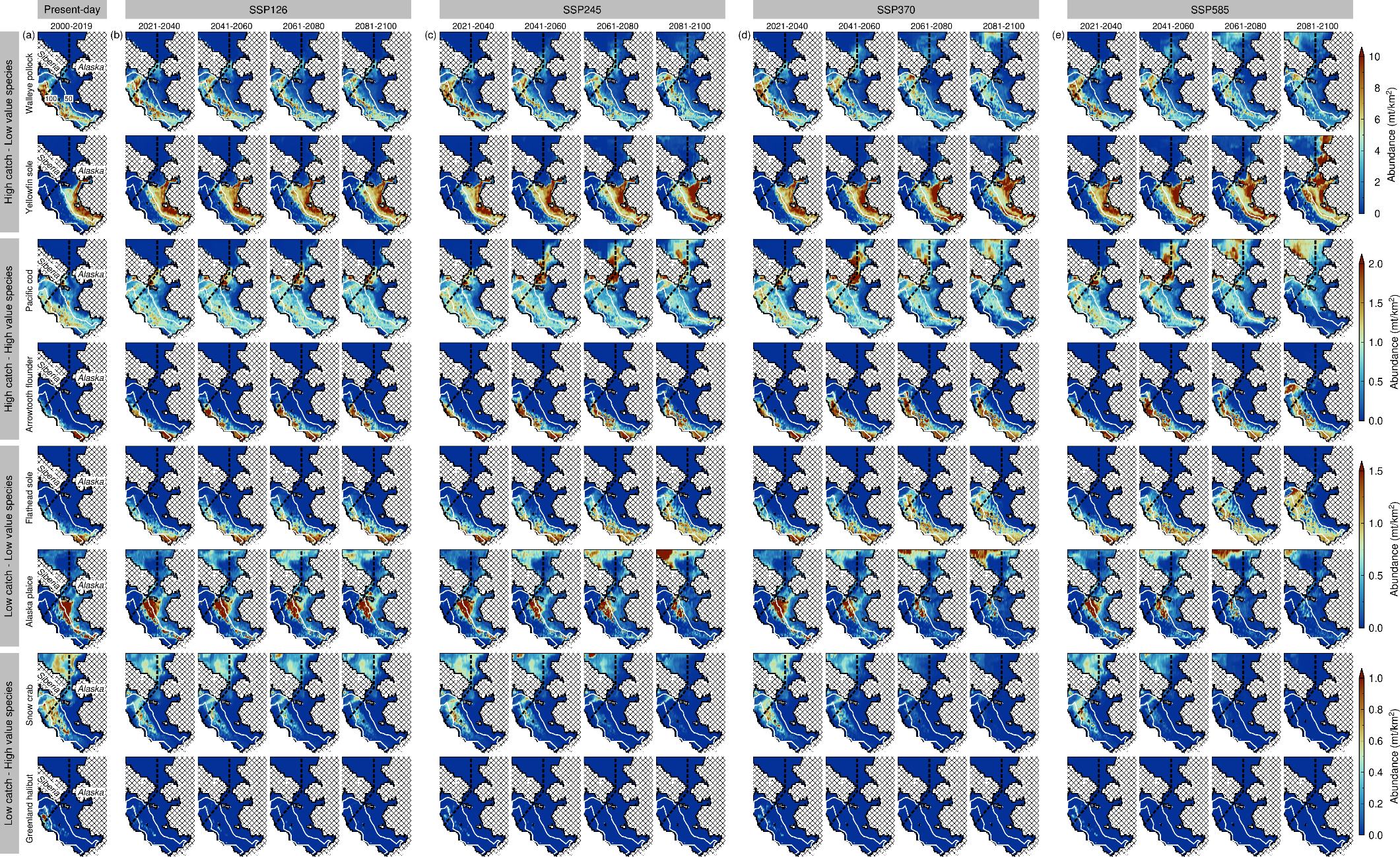
**

**Figure S8.** Spatial distributions of modeled abundance for (a) present (2000-2019) and future (2021-2100) periods under (b) SSP126, (c) SSP245, (d) SSP370, and (e) SSP585 for all species classified based on their catch and economic values. Overlain are the US-Russian border (dashed lines) and bathymetric contours (white solid lines) in the Eastern Bering and Chukchi seas.


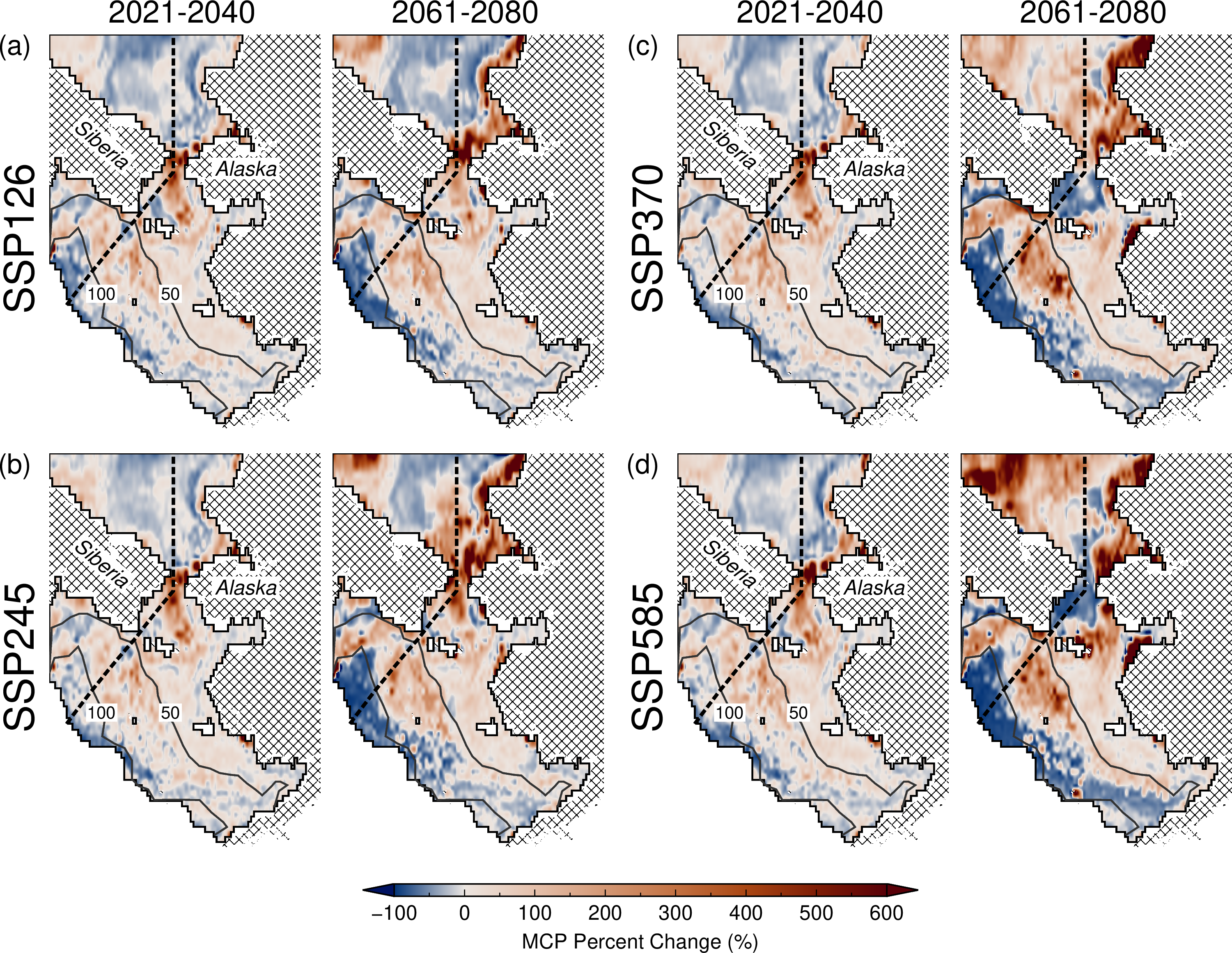


**Figure S9.** Predicted future (2021-2040; 2061-2080) percent changes in the cumulative maximum catch potential (MCP) under (a) SSP126, (b) SSP245, and (c) SSP370, and (d) SSP585 for eight major fisheries relative to the present (2000-2019). Overlain are the US-Russia EEZ boundary (dashed line) and bathymetric contours (solid gray lines).


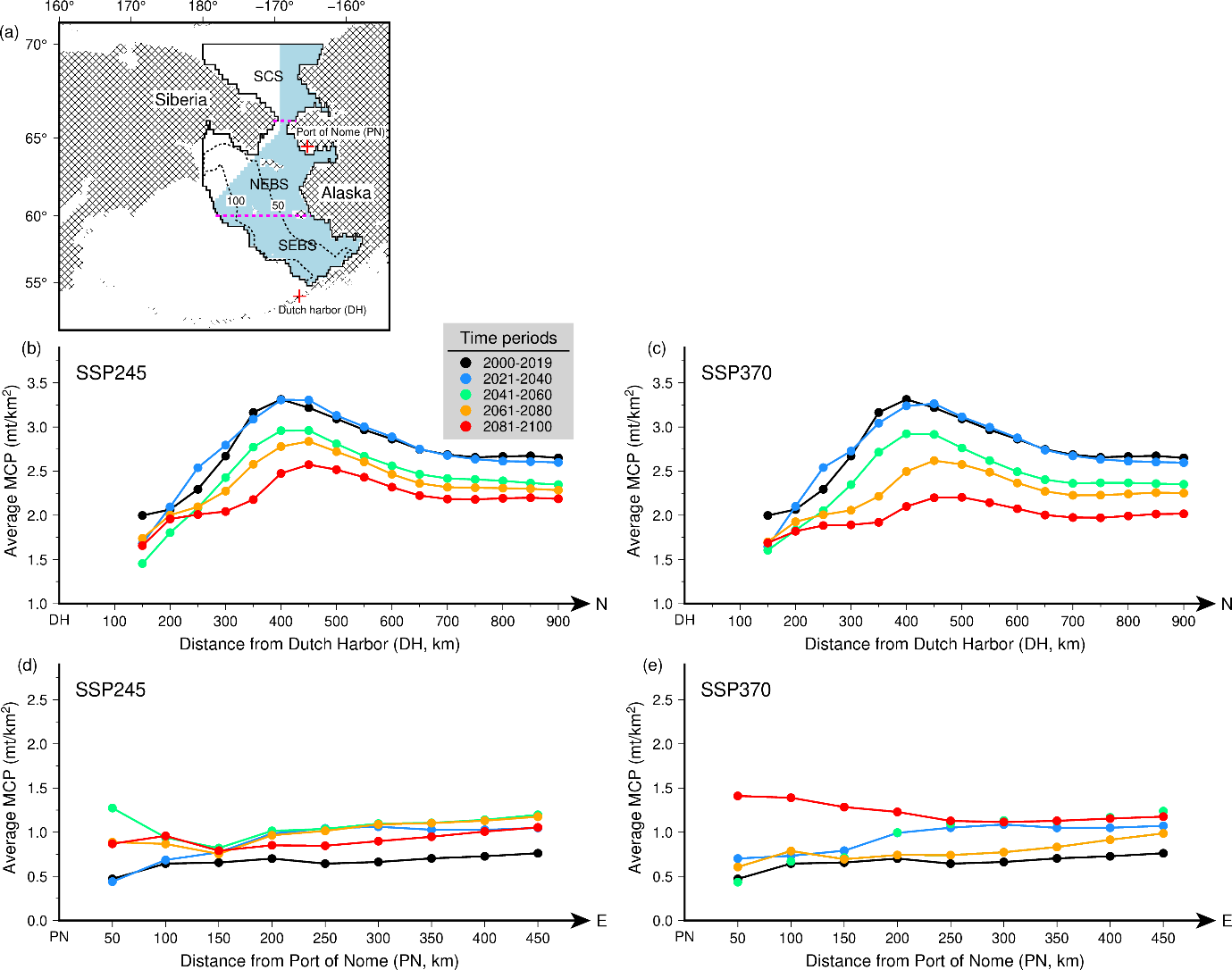


**Figure S10**. (a) Locations of the major and northern fishing ports (red crosses) in the Eastern Bering Sea and averaged MCP within the US EEZ (light blue polygon) computed at each 100-km buffer zone from the (b-c) Dutch Harbor and (d-e) Port of Nome for the present (2000-2019) and future periods (2021-2100) under SSP245 and SSP370. Bathymetric contours (black broken lines) and latitudinal (pink dashed lines) domains of the study area are shown in (a).


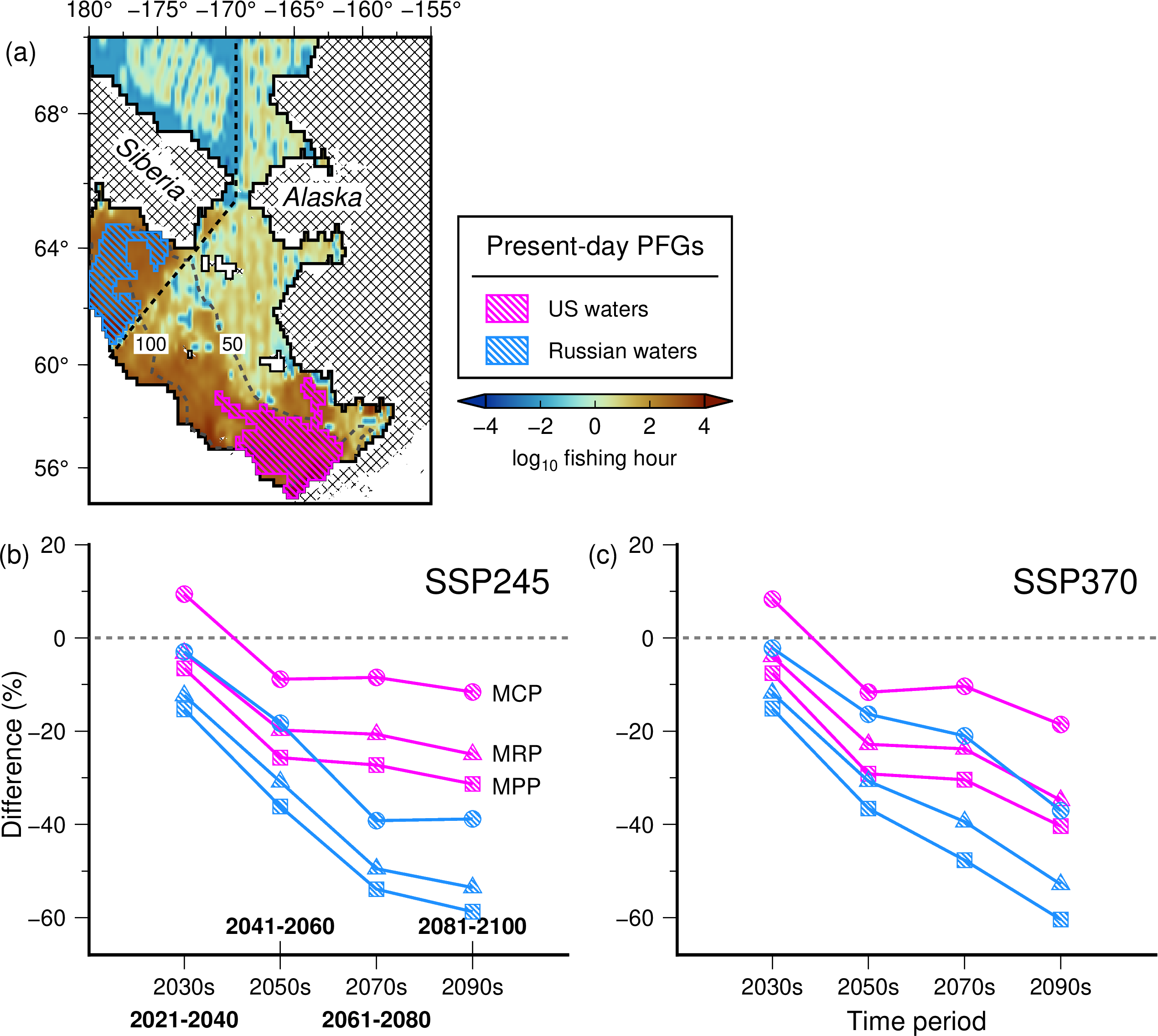


**Figure S11.** (a) Spatial distribution of aggregated fishing effort from 2012-2020, overlain with present-day potential fishing grounds (PFGs, fishing hour ≥ 1000 hrs). Differences between present (2000-2019) and future (2021-2100) maximum catch (circles), revenue (triangles), and profit (squares) potential for all species under (b) SSP245 and (c) SSP370 within the US (magenta shapes and lines) and Russian (blue shapes and lines) fishing grounds, respectively.

**References**

1. Gaines SD, Costello C, Owashi B, Mangin T, Bone J, et al. (2018) Improved fisheries management could offset many negative effects of climate change. Science Advances 4: eaao1378.

2. Costello C, Ovando D, Clavelle T, Strauss CK, Hilborn R, et al. (2016) Global fishery prospects under contrasting management regimes. Proceedings of the National Academy of Sciences 113: 5125-5129.

3. Ricard D, Minto C, Jensen OP, Baum JK (2012) Examining the knowledge base and status of commercially exploited marine species with the RAM Legacy Stock Assessment Database. Fish and Fisheries 13: 380-398.
